# GABAergic regulation of striatal spiny projection neurons depends upon their activity state

**DOI:** 10.1371/journal.pbio.3002483

**Published:** 2024-01-31

**Authors:** Michelle Day, Marziyeh Belal, William C. Surmeier, Alexandria Melendez, David Wokosin, Tatiana Tkatch, Vernon R. J. Clarke, D. James Surmeier

**Affiliations:** 1 Department of Neuroscience, Feinberg School of Medicine, Northwestern University, Chicago, Illinois, United States of America; 2 Department of Neurology, Baylor College of Medicine, Houston, Texas, United States of America; 3 Aligning Science Across Parkinson’s (ASAP) Collaborative Research Network, Chevy Chase, Maryland, United States of America; ICM - Institut du Cerveau et de la Moelle épinière Hôpital Pitié-Salpêtrière 47, bd de l’Hôpital, FRANCE

## Abstract

Synaptic transmission mediated by GABA_A_ receptors (GABA_A_Rs) in adult, principal striatal spiny projection neurons (SPNs) can suppress ongoing spiking, but its effect on synaptic integration at subthreshold membrane potentials is less well characterized, particularly those near the resting down-state. To fill this gap, a combination of molecular, optogenetic, optical, and electrophysiological approaches were used to study SPNs in mouse ex vivo brain slices, and computational tools were used to model somatodendritic synaptic integration. In perforated patch recordings, activation of GABA_A_Rs, either by uncaging of GABA or by optogenetic stimulation of GABAergic synapses, evoked currents with a reversal potential near −60 mV in both juvenile and adult SPNs. Transcriptomic analysis and pharmacological work suggested that this relatively positive GABA_A_R reversal potential was not attributable to NKCC1 expression, but rather to HCO3^-^ permeability. Regardless, from down-state potentials, optogenetic activation of dendritic GABAergic synapses depolarized SPNs. This GABA_A_R-mediated depolarization summed with trailing ionotropic glutamate receptor (iGluR) stimulation, promoting dendritic spikes and increasing somatic depolarization. Simulations revealed that a diffuse dendritic GABAergic input to SPNs effectively enhanced the response to dendritic iGluR signaling and promoted dendritic spikes. Taken together, our results demonstrate that GABA_A_Rs can work in concert with iGluRs to excite adult SPNs when they are in the resting down-state, suggesting that their inhibitory role is limited to brief periods near spike threshold. This state-dependence calls for a reformulation for the role of intrastriatal GABAergic circuits.

## Abstract 2

Synaptic transmission mediated by GABAA receptors in adult principal striatal spiny projection neurons can suppress ongoing spiking, but what is its effect on synaptic integration at sub-threshold membrane potentials? This study shows that GABA is a state-dependent neurotransmitter, and not simply an inhibitory neurotransmitter, suggesting a revision of the role of intrastriatal GABAergic signaling.

## Introduction

The striatum is the largest component of the basal ganglia circuitry regulating goal-directed actions and habits [1,2]. The principal neurons of the striatum are GABAergic spiny projection neurons (SPNs). SPNs integrate information arising from extrastriatal glutamatergic neurons, intrastriatal GABAergic interneurons, and collaterals of neighboring GABAergic SPNs. These intrastriatal GABAergic synapses, which constitute about 20% of all SPN synapses [3], and the postsynaptic GABA_A_Rs transducing the effects of synaptically released GABA, are widely viewed as inhibitory, working in opposition to dendritic excitatory glutamatergic input to suppress SPN spiking [4].

Although the ability of SPN GABA_A_Rs to suppress spiking is clearcut, characterizing them as categorically inhibitory fails to consider 2 salient features of adult SPNs. First, the reversal potential of GABA_A_Rs of SPNs appears to be relatively depolarized [5–7]. In particular, perforated patch recordings from relatively immature SPNs place the GABA_A_R reversal potential near −60 mV [6]. Indirect estimates of the GABA_A_R reversal potential have yielded similar values in more mature neurons [7,8]. Another key feature of SPN physiology that is relevant to the functional impact of GABA_A_Rs is their resting membrane potential. In the absence of synaptic input, the physiology of SPNs is dominated by somatodendritic Kir2 K^+^ channels, which drives the membrane potential to near the K^+^ equilibrium potential of roughly −80 mV [9]. In vivo, this so-called “down-state” in adult SPNs is interrupted by synaptically driven periods of depolarization when the somatic membrane transitions to potentials near −60 mV, close to spike threshold [10]. Although the synaptic determinants of these “up-states” in vivo are not well defined, ex vivo preparations have revealed that the depolarization arising from clustered glutamatergic synaptic activity on distal dendrites can drive regenerative, plateau potentials that mimic up-states [11–13]. A critical trigger of SPN dendritic plateau potentials is the engagement of N-methyl-D-aspartate receptors (NMDARs), which depends not only upon glutamate but also membrane depolarization and the displacement of pore-blocking Mg^2+^. This unblocking requires that the membrane depolarize to around −60 mV. Although recent work has shown that dendritic spikes in SPNs can be abbreviated by trailing GABAergic input at the site of glutamatergic stimulation [13], the roles of dendritic location, timing, and synaptic strength in determining the interaction between GABA_A_Rs and ionotropic glutamate receptors (iGluRs) have not been systematically explored in SPNs.

To better understand the role of GABA_A_R signaling in adult SPNs, a combination of experimental and computational approaches was employed. These studies revealed that juvenile and adult SPNs do not express significant levels of mRNA coding for NKCC1 but do express mRNA for KCC2 and HCO_3_^-^/Cl^-^ transporters. Perforated patch recordings from SPNs in ex vivo brain slices from mice over a broad range of ages (1 to 9 months) revealed that the GABA_A_R reversal potential was stable and near −60 mV. Thus, engagement of either synaptic or extra-synaptic GABA_A_Rs excited SPNs in the down-state, pushing them toward spike threshold. Furthermore, both experimental and modeling work demonstrated that leading dendritic GABA_A_R postsynaptic potentials (PSPs) effectively summed with trailing, spatially coincident iGluR-mediated depolarization. Moreover, computational studies revealed that when the GABAergic input was electronically remote from iGluRs, the 2 inputs effectively worked together to drive membrane depolarization regardless of timing. Given that SPNs in vivo appear to reside primarily at membrane potentials well below the reversal potential for GABA_A_Rs, these results suggest that physiological consequences of SPN GABAergic synapses should not be considered as simply inhibitory and that in a wide range of situations GABA_A_Rs work in concert with iGluRs to move SPNs closer to spike threshold, promoting their participation in network function.

## Results

### SPNs robustly expressed mRNA for KCC2, but not NKCC1

**Fig 1:**
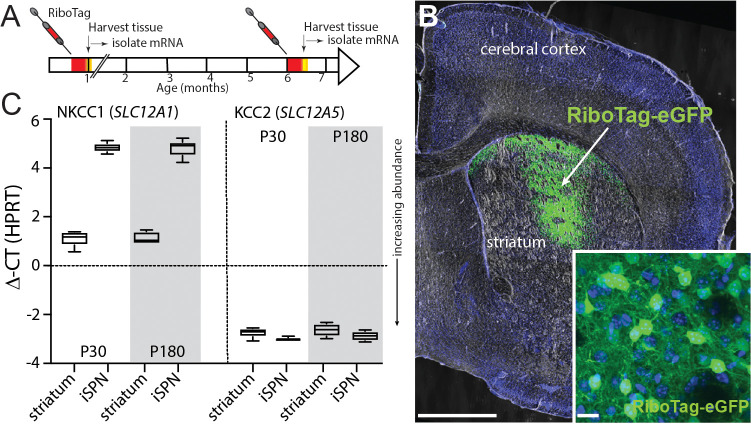
KCC2 mRNA is robustly expressed in SPNs, but not NKCC1. ** (A)** The RiboTag construct AAV5-DOI-hSyn-RpI22I1-3Xflag-2A-eGFP was injected into the striatum of Adora2a-cre mice at P18 or at 6 months of age. **(B)** The coronal slice images demonstrate both the coverage and restriction to the striatum of the stereotaxically injected AAV carrying the RiboTag and eGFP genes (stereotaxic injection coordinates: ML = −1.85, AP = +0.74, DV = −3.50). Scale bars = 1 mm and 20 μm. Ten days later, the infected tissue (green fluorescence) was dissected out with the aid of fluorescence microscopy and qPCR was performed. **(C)** mRNA abundance (ΔCT) levels for the chloride cotransporters NKCC1 (*SLC12A1*) and KCC2 (*SLC12A5*) were determined by qPCR in striata from Adora2a-cre mice 4 weeks and 6 months of age. The data underlying the graphs shown in the figure can be found in dx.doi.org/10.5281/zenodo.10386854. AAV, adeno-associated virus; qPCR, quantitative polymerase chain reaction; SPN, spiny projection neuron.

It is commonly thought that the reversal potential of GABA_A_Rs [14,15] is governed in large part by the balance between the plasma membrane cation/Cl^-^ co-transporters–NKCC1 and KCC2 [8,16]. To determine whether SPNs expressed NKCC1 and KCC2, the striata of 1- and 6-month-old *Adora2*-Cre mice were stereotaxically injected with an adeno-associated virus (AAV) carrying a DIO-RiboTag expression construct [17] **(Fig 1A)**. Four weeks later, mice were killed; total striatal mRNA and RiboTag-associated mRNA were harvested for quantitative polymerase chain reaction (qPCR) and RNASeq analyses **(Fig 1B)**. These experiments revealed that iSPNs robustly expressed mRNA coding for KCC2 (*Slc12a5*), but not NKCC1 (*Slc12a2*) **(Fig 1C)**. The relative expression of these transcripts did not change within the time window examined **(Fig 1C)**. The expression of RiboTag harvested, iSPN-specific transcripts was like the mRNA harvested from the entire striatum **(Fig 1C).**

### The GABA_A_R reversal potential was near −60 mV in both young and adult SPNs

**Fig 2:**
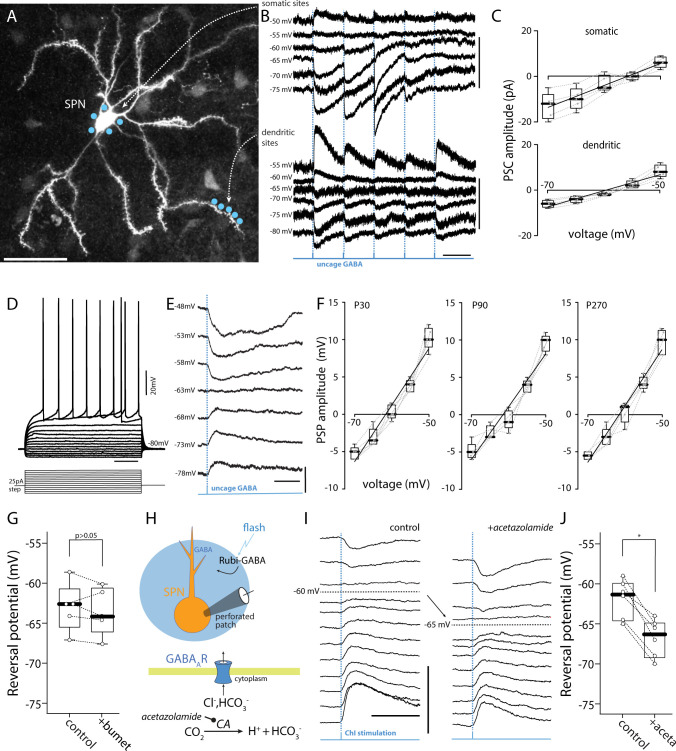
The GABA_A_R reversal potential is near −60 mV in both young and adult SPNs. **(A)** Clomeleon-expressing iSPNs allowed visual identification of dendrites in gramicidin perforated-patch recording conditions where cells cannot be loaded with dyes via internal-pipette solution (920 nm laser maximum projection image, scale bar = 40 μm). When low (<100 MΩ) access-resistance was achieved in voltage-clamp mode, RuBi-GABA (10 μM) was uncaged with a 473 nm laser spot (approximately 1 μm diameter, 1 ms) in the presence of the synaptic blockers: TTX (1 μM), AP5 (50 μM), NBQX (5 μM), CGP-55845 (1 μM). The laser was targeted to the somatic region or to distal dendrites (blue spots, projection image). **(B)** Representative voltage traces showing GABA responses, recorded in serial, from the soma (top traces, scale bars = 20 pA/2 s) or the dendrite (lower traces, scale bars = 10 pA/2 s) as the membrane was manually stepped from −70 mV to −50 mV. **(C)** Plot of the current/voltage relationship between somatic and dendritic activation. The data, represented by medians with interquartile ranges, did not differ significantly between the soma and dendritic compartments (*n* = 5 each; soma, dendrite; slope = 0.96, 0.71; x-intercept = −55.9, −59.6 mV; R^2^ = 0.77, 0.85, respectively). Current measurements were rounded to the nearest 0.5 pA. **(D)** Current-clamp experiments in gramicidin perforated-patch mode were performed to examine age-dependent shifts in reversal potential. Here, Adora2a-eGFP positive iSPNs could be visually identified and patched. When low (<100 MΩ) access-resistance was achieved in current-clamp mode, the resting membrane potential along with series of hyperpolarizing and depolarizing steps were used to examine cell health (traces, scale bars = 20 mV/200 ms). **(E)** RuBi-GABA (10 μM) was uncaged over the full-field (3 ms duration, 60× lens) with a 473 nm LED in the presence of the synaptic blockers: AP5 (50 μM), NBQX (5 μM), CGP-55845 (1 μM). Representative current traces showing GABA responses as the membrane was manually stepped from −80 mV to −50 mV, scale bars = 5 mV/200 ms. **(F)** Plot of the change in PSP amplitude at P30, P90, and P270. The data, represented by medians with interquartile ranges, did not differ significantly between the 3 ages tested (*n* = 5 each P30, P90, P270; slope = 0.75, 0.70, 0.75; x-intercept = −61.6, −61.5, −61.5 mV; R^2^ = 0.93, 0.91, 0.94, respectively). Values were calculated to the nearest 0.5 mV for the ΔV/V measurements. The data shows that the reversal for GABA-induced current is a full 20 mV+ above the resting membrane potential for SPNs, typically, −80 to −85 mV. **(G)** The addition of bumetanide (NKCC1 blocker, 10 μM) did not change the GABA_A_R reversal potential significantly (*n* = 5, *p* = 0.4076). **(H)** Perforated patch recordings were obtained from Adora2a-eGFP iSPNs and then the reversal potential of GABA_A_Rs determined before and after inhibition of CA with acetazolamide (aceta, 10 μM). **(I)** Representative traces recorded from a visually identified iSPN from an Adora2a-eGFP mouse in gramicidin perforated patch in current-clamp mode in the synaptic blockers: AP5 (50 μM), NBQX (5 μM), CGP-55845 (1 μM), MPEP (1 μM), and CPCCOEt (50 μM). RuBi-GABA (15 μM) was uncaged using a single LED pulse (470 nm, 25 ms). The pulse was applied at an interval of 30 s while manually stepping the cell to different potentials from −80 to −50 mV, scale bars = 10 mV/100 ms. **(J)** Summary data shows that application of acetazolamide shifted the reversal of the GABA-induced current to more negative potentials (*p* = 0.03125, *n* = 6). Membrane potentials were adjusted to correct for the estimated liquid junction potential and then binned into 5 mV increments (-70, -65, -60, -55 and -50 mV). The data underlying the graphs shown in the figure can be found in dx.doi.org/10.5281/zenodo.10386854. CA, carbonic anhydrase; PSP, postsynaptic potential; SPN, spiny projection neuron.

To determine the reversal potential of GABA_A_Rs in SPNs, ex vivo brain slices were prepared from young adult (6 to 7 months old) mice and then gramicidin perforated patch recordings were made from identified SPNs. Gramicidin is selectively permeable to monovalent cations, leaving the intracellular Cl^-^ concentration ([Cl^-^]_i_) unperturbed. To visualize dendrites, SPNs were sparsely labeled using an AAV carrying a SuperClomeleon expression plasmid [18] **(Fig 2A)**. To activate GABA_A_Rs, RuBi-GABA was uncaged on the soma and dendrites using a blue laser spot **(Fig 2A)**. The somatic membrane potential was clamped at membrane potentials between −50 and −70 mV prior to uncaging GABA and the resulting currents monitored (**Fig 2B**). The amplitude and polarity of uncaging evoked currents were then plotted as a function of somatic membrane potential. The estimated reversal potential for somatic GABA_A_Rs was near −55 mV **(Fig 2C)**. Dendritic uncaging of GABA evoked currents which also reversed in polarity near −60 mV **(Fig 2C)**.

To determine if there was any shift in the GABA_A_R reversal potential after weaning, gramicidin perforated patch recordings were made from SPNs in ex vivo brain slices taken from mice at 3 ages: young (approximately 1 month old), young adult (6 to 7 months old), and adult (approximately 9 months old) mice. SPNs recorded in this mode displayed the characteristic inward rectification, delayed time to the first spike at rheobase, and sustained repetitive spiking with suprathreshold current injection (**Fig 2D**). The membrane potential changes evoked in SPNs by RuBi-GABA uncaging on the peri-somatic membrane reversed near −60 mV at all ages (**Fig 2E and 2F**).

Why is the reversal potential of the GABA_A_Rs relatively depolarized? The striatal circuitry is largely quiescent in the ex vivo brain slice, making it highly unlikely that ongoing GABAergic signaling was loading neurons with Cl^-^ and pushing the reversal potential in a depolarized direction. Despite the absence of detectable levels of its mRNA, the functional contribution of NKCC1 to the reversal potential of GABA_A_R was tested by bath application of the NKCC1-selective antagonist bumetanide (10 μM); bumetanide did not change the GABA_A_R reversal potential (**Fig 2G**). Since GABA_A_Rs exhibit a significant permeability to HCO_3_^-^, the other determinant of the GABA_A_R reversal potential is the HCO_3_^-^ equilibrium potential [8]. To assess the role of intracellular HCO_3_^-^ in determining the GABA_A_R reversal potential, perforated patch recordings were obtained from SPNs in ex vivo brain slices (as described above) and then the reversal potential of GABA_A_Rs determined before and after inhibition of carbonic anhydrase (CA) with acetazolamide (10 μM). In vivo, CA catalyzes the conversion of cytosolic CO_2_ to H^+^ and HCO_3_^-^ (**Fig 2H**) [8]. RiboTag/RNASeq analysis revealed that iSPNs expressed 2 cytosolic CA subtypes with intracellular catalytic domains (*Car2>Car7; RNASeq read ratio = 4*.*3*)—in agreement with previous work [19]. Nonspecific inhibition of these CAs with acetazolamide led to a significant negative shift in the reversal potential of GABA_A_Rs (**Fig 2I and 2J**), consistent with the inference that the relatively depolarized GABA_A_R reversal potential in SPNs was attributable to HCO_3_^-^ flux.

### GABA_A_R engagement depolarized SPNs in the down-state

**Fig 3:**
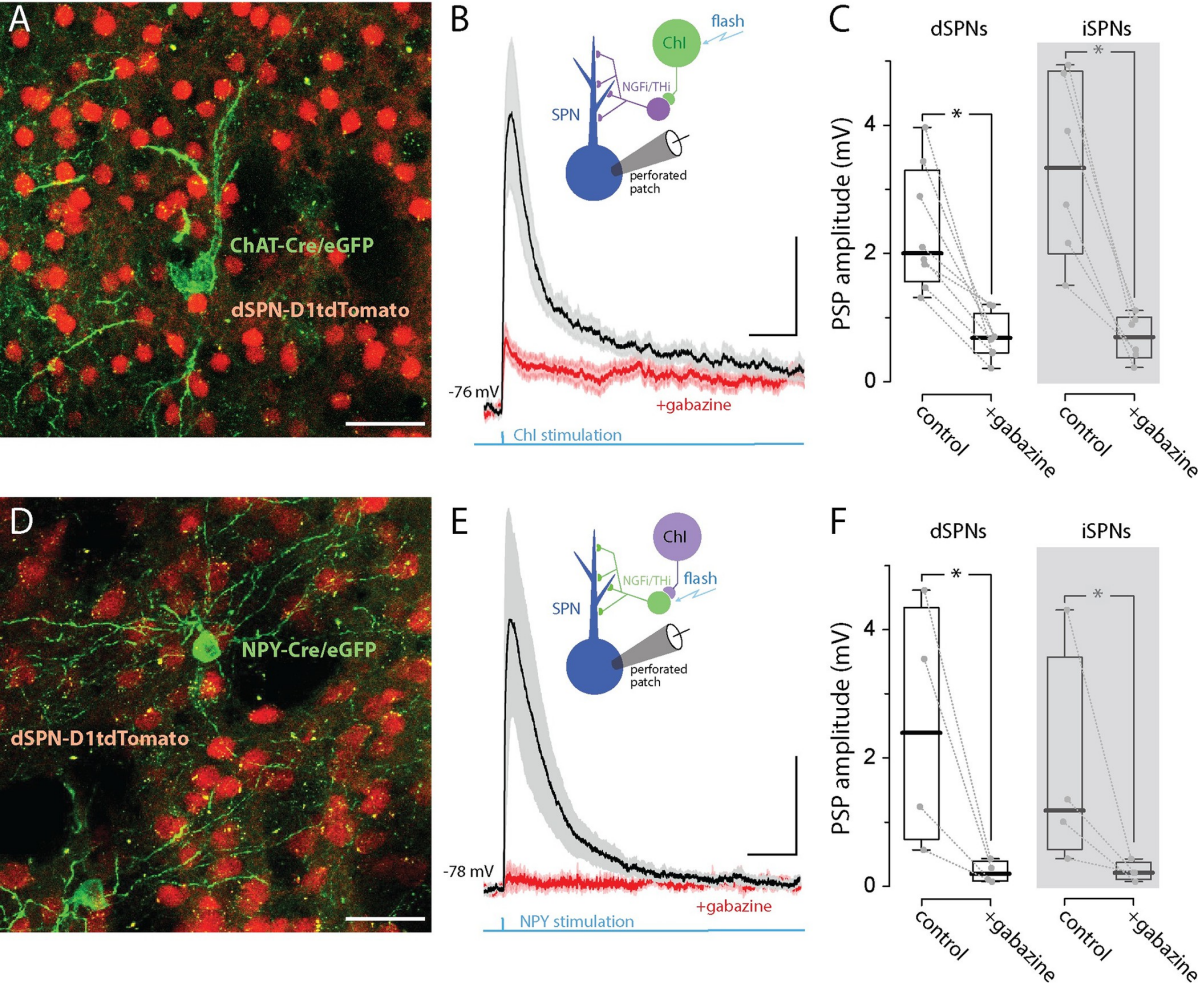
Optogenetic stimulation of ChIs or NPY-expressing interneurons evokes robust GABA_A_R-mediated PSPs in both iSPNs and dSPNs. **(A)** AAV9-hSyn-chronos-flex-eGFP was stereotaxically injected into the striatum of two-month-old ChAT-Cre X D1tdTomato mice (Stereotaxic coordinate injection: ML = −1.2, AP = −0.7, DV = −3.4). The coronal confocal slice image shows the expression of Chronos (green cells) in a ChAT-cre neuron (cholinergic interneurons) along with dSPNs expressing tdTomato (red cells, scale bar = 40 μm). The tissue was dissected and recorded from 21 days postinjection. **(B)** The mean (± SEM) of ChI-evoked EPSP responses recorded from visually identified SPNs in gramicidin perforated patch in current-clamp mode in the presence of synaptic blockers: NBQX (5 μM), AP5 (50 μM), CGP-55845 (1 μM), MPEP (1 μM), and CPCCOEt (50 μM). The LED pulse (470 nm, 5 ms) was applied at an interval of 60 s. The traces recorded before and after the addition of gabazine (10 μM). Scale bars = 1 mV/100 ms. **(C)** Box plots of data from dSPNs (*n* = 8) and iSPNs (*n* = 6). **(D)** NPY-Cre X D1tdTomato mice were injected as described in (A). Confocal image showing NPY-Cre neurons expressing Chronos (green) and dSPNs expressing tdTomato (red, scale bar = 40 μm). **(E)** Mean (+ SEM) of NPY-Cre-evoked EPSP responses recorded from visually identified dSPNs in gramicidin perforated patch in current-clamp mode in the presence of blockers as described in (B) before and after the addition of Gabazine (10 μM). Traces from dSPN recorded in NPY (*n* = 4). Scale bars = 1 mV/100 ms. **(F)** Summary data for dSPNs (*n* = 4) and for iSPNs (*n* = 4). The data underlying the graphs shown in the figure can be found in dx.doi.org/10.5281/zenodo.10386854. ChAT, choline acetyltransferase; ChI, cholinergic interneuron; PSP, postsynaptic potential; SPN, spiny projection neuron.

To study the role of synaptic GABA release, a mixed population of striatal GABAergic interneurons were activated by optogenetic stimulation of cholinergic interneurons (ChIs) [20]. Working through nicotinic acetylcholine receptors (nAChRs), ChIs can activate both neurogliaform interneurons (NGFIs) and tyrosine hydroxylase interneurons (THIs), giving rise to GABA_A_R-mediated currents in SPNs [21]. To monitor evoked responses in SPNs, perforated patch recordings were made from identified iSPNs or dSPNs using the approach described above. To optogenetically activate ChIs, an AAV carrying a Cre recombinase-dependent expression construct for Chronos was injected into the striatum of transgenic mice expressing Cre recombinase under the control of the choline acetyltransferase (ChAT) promoter (**Fig 3A**). In the ex vivo brain slice, SPNs are quiescent and reside in the down-state near −80 mV [10]. As predicted from the GABA uncaging studies above, optical stimulation of ChIs in the presence of iGluR antagonists evoked depolarizing, PSPs in SPNs that were blocked by the GABA_A_R antagonist gabazine **(Fig 3B and 3C)**.

To simplify the afferent circuitry engaged in these experiments, mice expressing Cre recombinase under the control of the NPY promoter (NPY-Cre) were injected with the same AAV vector used in the ChAT-Cre mice (**Fig 3D**). NPY is expressed by NGFis and low-threshold spike GABAergic interneurons (LTSIs) [4]—both of which make GABAergic synapses primarily on SPN dendrites [22]. Optogenetic activation of NPY-expressing interneurons alone produced depolarizing PSPs that were kinetically similar to those evoked by optogenetic stimulation of ChIs **(Fig 3E and 3F)**.

### GABA_A_R activation enhanced the depolarization produced by iGluRs

As shown previously, in both SPNs and pyramidal neurons [5,6,23], a depolarizing GABA_A_R input can boost the response to a trailing intrasomatic current injection and enhance the probability of spiking. However, GABA_A_R activation also can suppress spike generation by membrane shunting and pushing the membrane potential below spike threshold, which is typically between −45 and −50 mV [23].

How might the interaction between GABA_A_Rs and iGluRs play out in dendrites? A key feature of SPN dendrites beyond about the first major branch point (approximately 80 μm from the soma) is the ability to generate dendritic spikes or plateau potentials that can last for 50 to 200 ms [11–13,24]. These dendritic spikes require the temporal convergence of 10 to 15 glutamatergic inputs over a relatively short stretch (approximately 20 μm) of dendrite, which produces enough of a local depolarization to engage NMDARs and voltage-dependent Ca^2+^ channels. Previous experimental and modeling work has shown that opening GABA_A_Rs near the site of glutamatergic stimulation after spike initiation can truncate them, much like somatic situation described above. Indeed, as modeling suggests that the dendritic membrane potential during these spikes rises close to 0 mV, GABA_A_R opening should hyperpolarize the dendrites [13].

**Fig 4:**
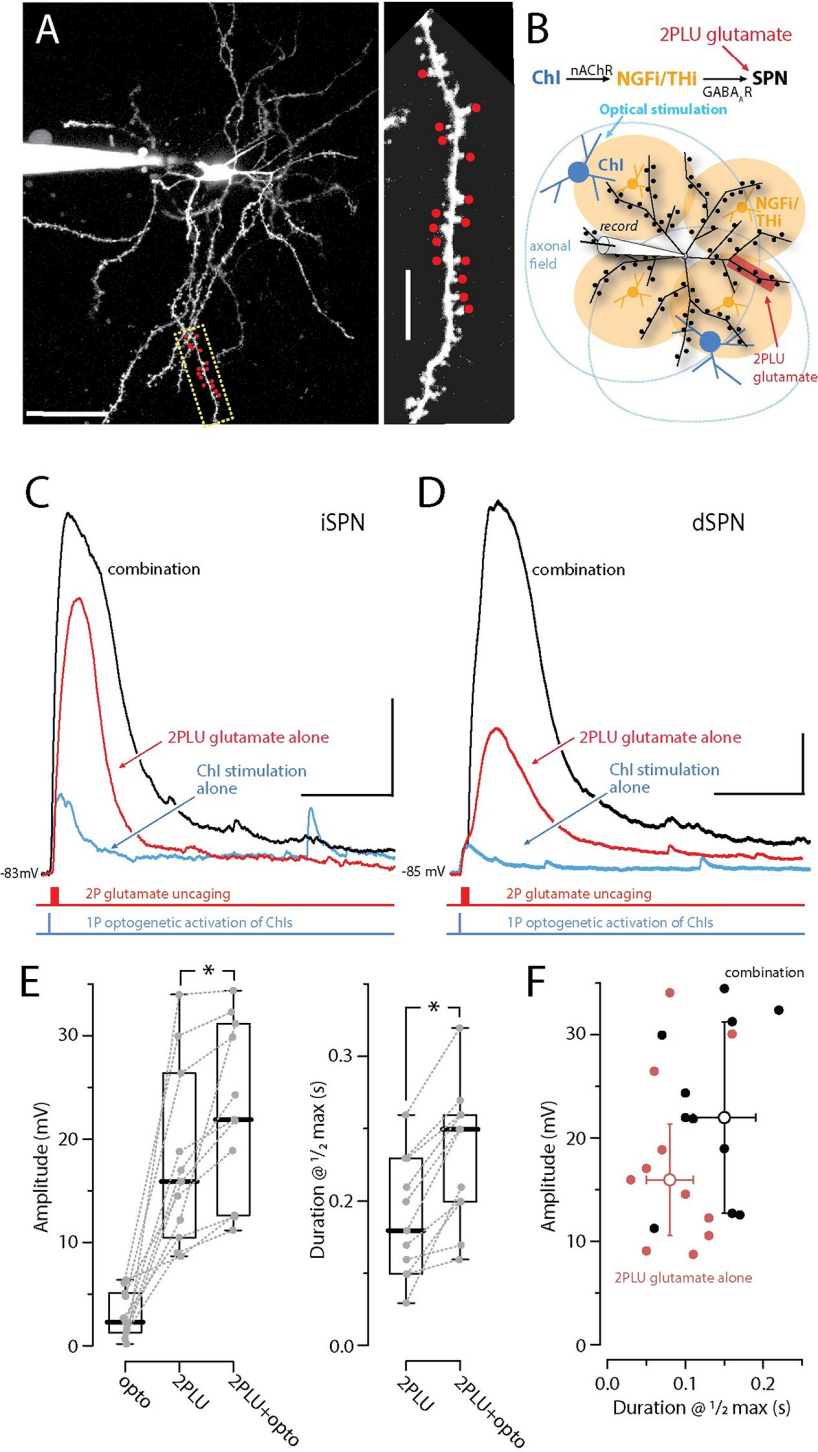
ChI-evoked stimulation of NGF interneurons and synaptic GABA release enhances glutamate-evoked state transitions. **(A)** Maximum projection image of a visually identified dSPN from a D1R-tdTomato x ChAT-cre mouse with a high magnification image of a distal dendrite where 720 nm 2PLSM spot uncaging of DNI-Glu (2PLU, 5 mM) was conducted (red dots). Tomato+ dSPNs were patched in whole-cell mode and the cells were loaded with Alexa 568 for clear identification of dendrites and spines. Scale bars = 40 μm cell, 5 μm dendrite. **(B)** Scheme for interrogating endogenous GABA release from NGFIs onto SPNs via optogenetic stimulation of ChAT-cre mice expressing Chronos. **(C, D)** Throughout the dendrites, glutamate uPSPs in dSPNs and iSPNs can be evoked by uncaging DNI-Glu (5 mM, 1 × 15 spines, 1 ms pulses at 500 Hz, red traces, 720 nm laser) while stimulating GABA release from NGFIs with the blue laser (1 × 3 ms pulse, blue traces, 473 nm, within approximately 20 μm of the dendrite). From the quiescent down-state, GABA_A_R activation is depolarizing and pushes SPNs toward enhanced dendritic integration in both dSPN and iSPN dendrites (Glu-2PLU + GABA_A_ opto = black trace, scale bars = 5 mV/200 ms). **(E)** Summary data showing the enhancement in amplitude and duration of the plateaus at ½ the maximum amplitude (1/2max) in iSPNs and dSPNs combined (*n* = 11 total: 3 iSPNs + 8 dSPNS; *p* **< 0.001** for both amplitude and 1/2max duration, respectively). **(F)** Scatter plot of duration at ½ maximum amplitude vs. amplitude for clustered glutamate alone (red) and following GABA_A_R activation (black). Median effects (open circles) and the median absolute difference as capped lines are also illustrated. All experiments are conducted in the appropriate cocktail of synaptic blockers: CGP-55845 (1 μM), MPEP (1 μM), and CPCCOEt (50 μM). The data underlying the graphs shown in the figure can be found in dx.doi.org/10.5281/zenodo.10386854. 2PLSM, two-photon laser scanning microscopy; ChAT, choline acetyltransferase; ChI, cholinergic interneuron; SPN, spiny projection neuron.

But, what if the GABA_A_R activation precedes the glutamatergic input to dendrites? A priori, one might predict that the dendritic depolarization produced by GABA_A_R opening would enhance the response to trailing glutamatergic input, much like the situation described at the soma. To test this hypothesis, 2 sets of experiments were performed. Identified iSPNs or dSPNs were recorded from in whole-cell mode to allow them to be filled with a dye (Alexa 568) and imaged using two-photon laser scanning microscopy (2PLSM) [25]. The [Cl^-^] in the pipette was adjusted to yield a GABA_A_R reversal potential near −60 mV. Next, a region of parfocal dendrite was identified to allow two-photon uncaging of DNI-glutamate at visualized spine heads [11,26,27]. In the first set of experiments, dendritic GABA_A_Rs were activated by optogenetic stimulation of ChIs as described above. Because of their large axonal field and those of the NGFIs/THIs they activate [4,28], optogenetic stimulation of ChIs should produce a diffuse GABAergic input to the dendrites of the recorded SPN **(Fig 4A and 4B)**. As shown above, optogenetic stimulation of ChIs alone evoked a consistent but modest somatic depolarization **(Fig 4C and 4D)**. Dendritic uncaging of glutamate alone also evoked a somatic depolarization. The number of axospinous sites stimulated was adjusted to be subthreshold for dendritic spike generation (assessed by the decay of membrane potential after termination of uncaging) **(Fig 4C and 4D)**. When this uncaging event was preceded by ChI-evoked GABA_A_R depolarization, the resulting magnitude and duration of the somatic depolarization was significantly increased in both types of SPN **(Fig 4E)**. A scatter plot of the algebraic sum of the amplitudes of the GABAergic and glutamatergic PSPs in isolation against the amplitude of the response to the combined stimulation revealed that the 2 inputs almost invariably summed linearly or supra-linearly (**S1 Fig**). A scatter plot of the amplitude and duration of iGluR-mediated responses demonstrated that prior engagement of GABAergic interneurons (by ChI stimulation) enhanced the iGluR-mediated responses (**Fig 4F**). Thus, transiently opening dendritic GABA_A_Rs produced a dendritic membrane potential change that enhanced the ability of subsequent dendritic glutamatergic input to push SPNs toward the local spike threshold.

### Computational modeling of dendritic integration in SPNs

**Fig 5:**
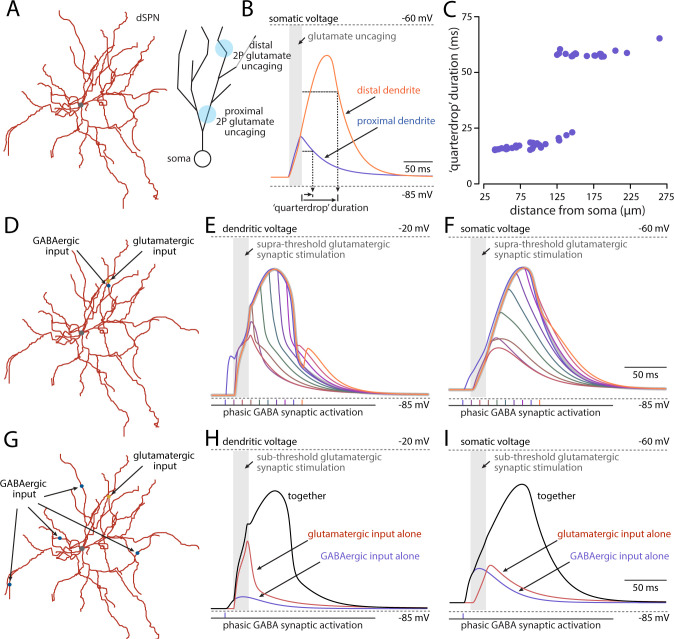
Computational modeling of dSPN dendritic activity. **(A)** Morphology of reconstructed dSPN with cartoon to illustrate the direction of stimulation along a dendrite. **(B)** Synaptic potentials recorded at the soma in response to clustered spine activation (18 neighboring glutamatergic synapses stimulated sequentially at 1 ms intervals) in 2 separate dendrites. A dendritic spike is generated to the distal (orange trace) but not proximal dendritic input (blue trace). The quarterdrop duration is defined as the time interval between the last stimulation and the time for the membrane voltage to drop by one quarter of its peak value. **(C)** Quarterdrop interval (ms) plotted as a function of path distance from the center of that dendrite to the soma (μm) for every dendrite with spines (50) of the reconstructed dSPN. Dendritic spikes were only reliably observed in distal dendrites (>100 μm from cell soma). **(D)** Onsite phasic GABAergic activation and glutamatergic activation delivered to the same distal dendrite. Synaptic potentials recorded at the dendrite **(E)** and soma **(F)**. GABA synaptic activation comprised 3 simultaneous stimulations delivered 5 times at an interval of 1 ms to the midpoint of the dendrite. The timing of this phasic input was varied relative to a fixed clustered supra-threshold glutamatergic input (delivered to 18 spines; 1 ms interval as before) in intervals of 10 ms from −10 (blue) to 80 ms (orange). For comparison, the effect of glutamatergic activation alone is illustrated by a thick gray line. Onsite GABAergic activation causes a dramatic cessation of dendritic and somatic potentials in a manner consistent with the relative timing of the 2 inputs. **(G)** Offsite phasic GABAergic activation delivered to 4 distal dendritic locations. A clustered glutamatergic input (15 spines; 1 ms interval) delivered to the same dendrite as before resulted in a subthreshold synaptic potential (red) at the dendritic site of delivery **(H)** and soma **(I)**. Similarly, the effect of only activating GABAergic synapses at 1 ms intervals at each of the 4 offsite dendritic locations simultaneously (3 per dendrite; 12 in total) resulted in a moderate postsynaptic potential (blue trace). When delivered sequentially, with GABAergic activation preceding glutamatergic by 10 ms, the previously subthreshold glutamatergic input (red traces) resulted in the generation of a spike (black trace; **H** and **I**). The data underlying the graphs shown in the figure can be found in dx.doi.org/10.5281/zenodo.10386854. SPN, spiny projection neuron.

Although intriguing, the experimental results presented are limited by the inability to control the timing and location of GABAergic input to dendrites in a rapid precise manner. Understanding how the timing and dendritic location of GABA_A_R activation modulates the response to clustered excitatory input could provide insight into the role of GABAergic interneurons in striatal computation. To help achieve a better grasp of the mechanisms underlying this interaction, a modified NEURON model of a dSPN [13,29–31] was used to assess the impact of timing and location of GABAergic input on the response to clustered glutamatergic synaptic input to a stretch of distal dendrite. As observed experimentally, clustered glutamatergic input was able to generate NMDAR-dependent, dendritic spikes or plateau potentials when delivered to distal dendrites of a quiescent neuron (**Fig 5A–5C**).

When GABAergic synapses were activated near glutamatergic synapses, the model behaved as previously described by Du and colleagues. That is, GABAergic input at almost any point during the dendritic spike (when the local membrane potential was near −30 mV) led to inhibition of both the dendritic and somatic membrane potential (**Fig 5D–5F**). However, the impact of GABAergic input was very different when the site of stimulation was at some distance from that of glutamatergic stimulation. For example, if the GABAergic input was distributed at distal locations across the dendritic tree, as predicted to happen following ChI or NGFI/THI activation, the effect was consistently excitatory. To illustrate this point, the distributed GABAergic input was followed by a subthreshold dendritic glutamatergic input. In this scenario, the combination of GABAergic and glutamatergic input led to an NMDAR-dependent dendritic spike (**Figs 5G–5I and S4A–S4C**)—just as seen experimentally using optogenetic stimulation of GABAergic interneurons and 2P uncaging of glutamate.

The default value for cytoplasmic resistivity (R_a_) in these computational simulations was 200 Ω cm. However, R_a_ is a difficult parameter to measure experimentally and there is little overall consensus as to its true value and estimates vary at least 5-fold (70 to 350 Ω cm) [32]. For this reason, simulations were repeated with R_a_ set to 100 Ω cm. The results were qualitatively similar to those described above (**S2 Fig**), suggesting that R_a_ is not a major factor in our simulations when varied within the proposed physiological range.

**Fig 6:**
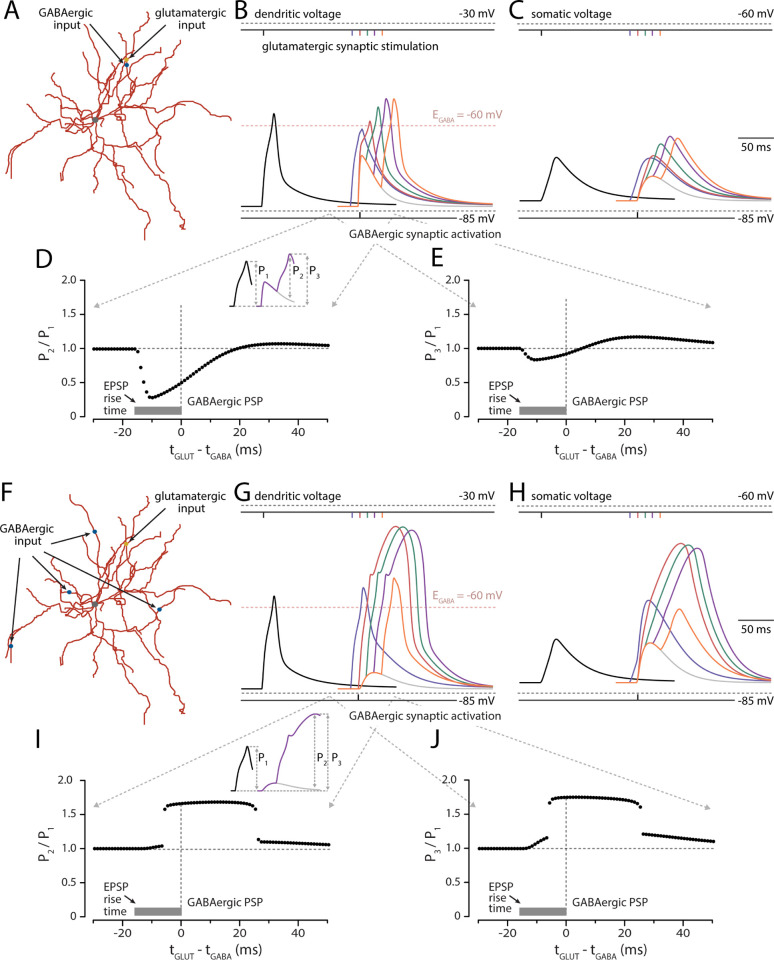
The interaction between glutamatergic and GABAergic synaptic activity. **(A)** On-site phasic GABAergic activity was delivered to the same dendrite as glutamatergic synaptic input. Synaptic potentials are illustrated at **(B)** dendritic site of glutamatergic activity and **(C)** cell soma. The black trace represents the effect of glutamate-mediated excitation alone (15 neighboring spines activated at 1 ms intervals along the chosen dendrite). The light gray trace illustrates the effect of phasic GABAergic stimulation (3 simultaneous synaptic stimulations delivered 4 times at an interval of 1 ms to the same dendritic site giving a total of 12 GABA synapses activated). Color traces represent the effect of varying glutamatergic stimulation at temporal intervals relative to the fixed GABAergic input described (blue to orange illustrate 5 traces with Δ**t** = t_GLUT_−t_GABA_ in the range of −10 to 30 ms, respectively, at 10 ms intervals). Inset illustrates the measurement of P_1_, P_2_, and P_3_. The absolute amplitude of the synaptic potential in the dendrite was measured in the absence of GABAergic activity (P_1_) or either relative to the amplitude of the underlying depolarizing phasic GABAergic potential at the peak of the **postsynaptic** response (P_2_) or relative to the underlying baseline (P_3_). **(D)** and **(E)** show the sublinear effect of varying glutamatergic spine activation relative to a fixed onsite GABA synaptic input on P_2_ and P_3_ normalized to P_1_, respectively. **(F)** Off-site phasic GABAergic activity was delivered to 4 distal dendrites distinct from the dendrite receiving clustered spine excitation. As before, **(G)** and **(H)** show simulated synaptic potentials recorded at dendrite and soma. The black trace represents the same glutamatergic input as above (i.e., 15 spines activated at 1 ms intervals). The light gray trace represents the effect of phasic GABAergic activity delivered to the 4 distal dendrites at the same time (as 3 synaptic simulations at 1 ms intervals per dendrite giving a total of 12 GABA synapses activated). Again, as before, color traces show the effect of altering the timing of clustered spine activation relative to GABA activity. In contrast to on-site activity, Δ**t** = 0, 10, and 20 ms results in the generation of a dendritic spike. Note that the peak of the EPSP_GLUT_ is still clearly visible before the trailing spike manifests. **(I)** and **(J)** illustrate the supralinear effect of varying glutamatergic spine activation relative to a fixed off-site GABA synaptic input on P_2_ and P_3_ normalized to P_1_, respectively. The data underlying the graphs shown in the figure can be found in dx.doi.org/10.5281/zenodo.10386854.

In many of the previous studies examining the impact of GABA_A_Rs on dendritic integration of glutamatergic input, the focus has been on the role of timing and location dependent GABA_A_R -mediated shunting of iGluR-evoked EPSPs on the same dendrite [33–35]. As shown above, our results are largely consistent with this literature. Of particular interest is the timing dependence of the interaction. To explore this relationship in SPNs, NEURON simulations were run with on-site glutamatergic and GABAergic input to the same distal dendrite (**Fig 6A**). As expected, there was a strong timing dependence on the interaction between synaptic events. As experimentally shown by others [6,23], when the glutamatergic EPSP preceded a neighboring GABAergic input, the effect of opening GABA_A_Rs on the input impedance (i.e., shunting) was clearly evident (**Fig 6B and 6C**). However, when the glutamatergic input arrived later, there was synaptic summation, albeit sublinear at both dendritic (**Fig 6B**) and somatic locations (**Fig 6C**). To better illustrate the quantitative interaction between the 2 inputs at the dendritic site of stimulation, 2 plots were generated. In one, relative amplitude of glutamatergic EPSP (P_2_) with a concomitant GABAergic input was divided by the amplitude of the glutamatergic EPSP alone (P_1_) and then plotted as a function of the relative timing of the 2 inputs (**Fig 6D**). This ratio (P_2_/P_1_) fell when the glutamatergic input preceded the GABAergic input and then rose when it trailed the GABAergic input. Similarly, if the ratio of the peak amplitude of the aggregate potential (P_3_) was divided by the peak amplitude of the isolated glutamatergic EPSP (P_1_) and plotted as a function of the relative timing of the 2 inputs, the ratio fell when the glutamatergic input preceded the GABAergic input, but then rose above 1 when the glutamatergic trailed the GABAergic input (**Fig 6E**).

Of greater interest, given the architecture of striatal circuits, was how a diffuse GABAergic input (mimicking conditions produced by ChI activation) would affect the interaction between synaptic events. To explore this interaction, the temporal relationship between a focal glutamatergic input to a distal dendrite and a GABAergic input to 4 neighboring dendrites was examined (**Fig 6F**). Not surprisingly, in this situation there was no shunting and the 2 inputs summed at both the dendritic (**Fig 6G**) and somatic (**Fig 6H**) locations—regardless of relative timing. In fact, GABAergic input enhanced the ability of glutamatergic synapses to trigger a dendritic spike **(Fig 6G and 6H)**. To probe the dendritic interaction, the relative amplitude of the mixed PSP (measured relative to the underlying GABAergic PSP—P_2_) was divided by the amplitude of the isolated glutamatergic EPSP (P_1_) and then plotted as a function of the relative timing of the 2 inputs. At all intervals, the ratio was greater than or equal to 1 (**Fig 6I**). A qualitatively similar plot was obtained by computing the ratio of the peak amplitude of the mixed PSP (P_3_) divided by the glutamatergic EPSP amplitude (P_1_) as a function of relative timing of the 2 inputs (**Fig 6J**).

In these simulations, 3 GABAergic synapses were activated in sequence (1 ms interval) at 4 distal, off-path dendrites (a total of 12 GABAergic synapses activated). Increasing the number of dendrites stimulated to 12, each receiving 1 GABAergic synapse (i.e., to maintain a total of 12 synapses activated) produced qualitatively similar results (**S4D–S4H Fig**). The widening of the temporal window for supralinear summation presumably resulted from an increase in amplitude of the GABAergic PSP at the site of glutamatergic input. In this simulation, 9 (or more) GABAergic synapses were needed for supralinear summation (**S4I and S4H Fig**). When the location of GABAergic synapses was shifted to a more proximal dendritic location, shunting decreased (**S3A–S3C Fig**) and the temporal window for supralinear summation broadened (**S3D and S3E Fig**). Thus, only GABAergic synapses near the site of glutamatergic input prevented supralinear summation. However, if a dendritic spike was generated, proximal GABAergic input had little effect on its propagation to the soma.

**Fig 7:**
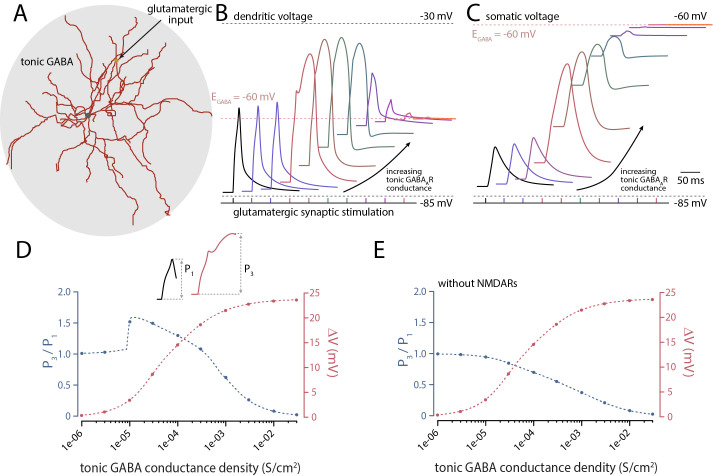
The interaction between tonic GABA_A_R signaling and glutamatergic activity. **(A)** Tonic GABAergic signaling was modeled as a conductance that was evenly distributed throughout the soma and dendritic tree. Glutamatergic synaptic input was delivered to the dendrite illustrated as clustered spine excitation as before. Synaptic potentials at the dendritic site of glutamatergic input **(B)** and at soma **(C)** are illustrated. The black trace represents the effect of synaptically released glutamate alone (15 neighboring synapses excited at 1 ms intervals). Color traces (blue to red) illustrate the effect of increasing tonic GABA conductance (from 10^−6^ to 3 × 10^−2^ S/cm^2^) on the glutamate response and resting membrane potential. Inset of **(D)** illustrates the measurement of P_3_ relative to P_1_ with absolute amplitudes measured relative to its underlying resting membrane potential. **(D)** Increasing tonic GABA conductance density caused an incremental depolarization (ΔV in red) that approached the reversal potential for E_GABA_ (−60 mV) at >10^−2^ S/cm^2^. Increasing depolarization (2–3 mV to 10^−5^ S/cm^2^ density) was accompanied by a dendritic spike in response to a glutamatergic input that was subthreshold in the absence of tonic GABA activation. This supralinear summation persisted for greater than an order of magnitude increase in tonic GABA activation (3 × 10^−4^ S/cm^2^); larger values led to local shunting. **(E)** Illustrates the requirement for glutamate-mediated synaptic activation of NMDAR for tonic GABA-mediated spike generation. The simulation was identical to that illustrated in (B–D) except that the NMDAR conductance at glutamatergic synapses was zero. In the absence of NMDARs, a combination of onsite shunt and reduced AMPAR driving force arising from depolarization presumably underlies the GABA conductance density-dependent decrease in response. The data underlying the graphs shown in the figure can be found in dx.doi.org/10.5281/zenodo.10386854. NMDAR, N-methyl-D-aspartate receptor.

SPNs exhibit tonic GABA_A_R-mediated currents [36]. To assess its effect on dendritic integration, tonic GABA_A_R current was modeled as a uniformly distributed conductance (**Fig 7A**). As expected, increasing tonic GABA_A_R conductance density progressively depolarized the membrane potential, approaching the reversal potential for E_GABA_ (−60 mV) (**Fig 7B and 7C**). A modest elevation in the tonic GABA_A_R current led to a dendritic spike in response to a previously subthreshold, clustered excitatory input. These spikes, like those described previously (**S4A–S4C Fig**), were dependent upon NMDARs (**Fig 7E**). The excitatory effect of tonic GABAergic currents on dendritic excitability was evident over a broad range of conductance values, with shunting becoming significant only at large values (**Fig 7D**). Thus, the “dose-response” relationship between tonic GABA_A_R current and “boosting” of the dendritic response to glutamatergic input had an inverted “U” shape.

**Fig 8:**
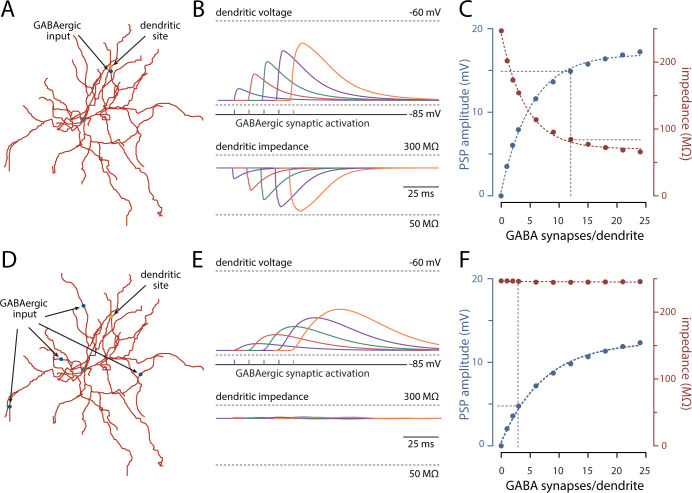
On vs. off-site GABAergic activation and local input impedance. **(A)** On-site phasic GABAergic activity was delivered and recorded in the same dendrite. **(B)** Color traces (blue to orange; above) represent simulated GABAergic PSPs and local dendritic impedance (to 10 Hz; below) to increasing synaptic activation (1, 3, 6, 12, and 24 GABA synapses per dendrite). **(C)** Increasing PSP amplitude (blue) to GABAergic synaptic stimulation reduced local impedance (red). **(D)** Off-site phasic GABAergic activity was delivered to 4 distal dendrites and recorded in the same dendrite as in (A). **(E)** and **(F)** as above. Note the lack of effect of increasing off-site GABAergic activity on local impedance. The dotted lines on panels (C) and (F) illustrate the effect of activating 12 GABAergic synapses in total, which represents the common scenario setting for these 2 conditions throughout the study. The data underlying the graphs shown in the figure can be found in dx.doi.org/10.5281/zenodo.10386854. PSP, postsynaptic potential.

To better illustrate the role of location in dictating the shunting effect of a GABAergic synapse, the dendritic voltage and input impedance at the distal dendritic site was computed for a range of GABAergic synapses/dendrite (0–24). Near the site of GABAergic input, the evoked dendritic depolarization progressively increased with the number of GABAergic synapses, but the input impedance fell in parallel (**Fig 8A–8C**). In contrast, on neighboring dendrites, the depolarization grew with the number of synapses, but there was little local change in input impedance (**Fig 8D–8F**).

The inference to be drawn from these simulations is that the interaction between dendritic glutamatergic and GABAergic synapses depends upon both location and timing. When co-localized, the timing of the 2 inputs dictates their interaction, as previously described [6,23]. However, when the GABAergic input is more diffuse, as predicted to occur with activation of ChIs or NGFIs, the relative timing of GABAergic and glutamatergic inputs becomes less important, and the 2 depolarizing inputs sum. This additivity allows GABAergic and glutamatergic synapses to work together to trigger dendritic spikes—as observed experimentally.

## Discussion

There are 2 main conclusions that can be drawn from the data presented. First, the GABA_A_R reversal potential in mature striatal SPNs is near −60 mV. This relatively depolarized reversal potential was largely attributable the HCO_3_^-^ permeability of GABA_A_Rs [8,15]. Second, in resting SPNs residing near the K^+^ equilibrium potential (approximately −80 mV), engagement of striatal GABAergic interneurons produced a depolarizing PSP much like that generated by iGluRs—in contrast to the inhibitory effect on SPNs near spike threshold. Both experimental and computational studies of resting SPNs demonstrated that GABAergic synapses could promote dendritic spike generation in response to glutamatergic input in a variety of biologically relevant situations. Taken together, these results argue that SPN GABA_A_R signaling should be considered as state-dependent and not strictly inhibitory or excitatory [8,15,36,37].

### GABA_A_R signaling in resting SPNs was excitatory

Based upon perforated patch recordings, the SPN GABA_A_R reversal potential remained relatively depolarized from the earliest time point examined (post-weaning) to adulthood. Based upon the analysis of mRNA harvested from iSPNs (using the RiboTag method), it can be concluded that mature SPNs robustly expressed KCC2, but not NKCC1, after weaning. The low level of NKCC1 expression in iSPNs was not due to our detection methodology, as at the tissue level, NKCC1 mRNA was readily seen. Moreover, NKCC1-selective antagonist bumetanide had no effect on the GABA_A_R reversal potential. Although it is possible that SPNs expressed NKCC1 at embryonic or pre-weaning stages of development, NKCC1 was not a determinant of SPN GABA_A_R function in mice post-weaning.

What then is the explanation for the depolarized reversal potential of GABA_A_Rs in SPNs? SPNs expressed mRNA for 2 isoforms of CA with cytosolic catalytic sites capable of converting intracellular CO_2_ to H^+^ and HCO_3_^-^. Inhibiting CAs with acetazolamide led to a significant negative shift in the GABA_A_R reversal potential in perforated patch recordings. Thus, CA mediated regulation of intracellular pH and HCO_3_^-^ contributed to the depolarized SPN GABA_A_R reversal potential [8,38].

One of the distinctive physiological features of SPNs is that at rest, constitutively active, inwardly rectifying Kir2 K^+^ channels control the transmembrane potential, pulling SPNs close to the K^+^ equilibrium potential near −80 mV—the so-called “down-state” of SPNs [10,39]. As these channels are distributed throughout the somatodendritic membrane, they also are a major determinant of local input resistance and dendritic electrotonic structure [3]. With depolarization intracellular Mg^2+^ ions and polyamines are swept into the channel pore blocking it [40]. Thus, in dendritic regions of SPNs in the down-state, GABA_A_R activation will depolarize the membrane and, in so doing, shut off Kir2 channels. As the depolarization produced by transient opening of synaptically activated GABA_A_Rs outlasts the change in input impedance, dendritic GABA_A_R activation should not only depolarize dendrites but increase their input impedance. Even in the case of tonic GABA signaling, GABA_A_R opening and Kir2 K^+^ channel block will counteract one another producing less of a change in dendritic input impedance than would have been predicted otherwise.

Although in principle, GABA_A_R signaling in SPN dendrites should enhance the ability of glutamatergic synapses to promote spike generation, this has never been directly tested with synaptic stimulation. Previous work on this interaction in ex vivo brain slices used intrasomatic current injection to demonstrate the interaction [6]. To fill this gap, optogenetic tools were used in conjunction with two-photon uncaging of glutamate at visualized dendritic spines. Optogenetic stimulation of ChIs was used to engage intrastriatal GABAergic interneurons that synapse on SPN dendrites [20]. Indeed, optogenetic stimulation of ChIs evoked a robust gabazine-sensitive PSP in SPNs recorded in perforated patch mode, as did direct optogenetic stimulation of NPY-positive GABAergic interneurons. In both cases, the GABAergic PSP was considerably delayed and slower than those evoked by SPN collaterals or fast-spiking interneurons [4,22]; most likely, this reflects the large axonal arbor of ChIs and NGFIs and the resulting diffuse release of GABA over the SPN dendritic tree.

To probe the interaction between this diffuse GABAergic input and focal activation of glutamatergic synapses, two-photon laser scanning uncaging of glutamate along a parfocal stretch of distal dendrite was used in conjunction with optogenetic stimulation of ChIs. Spatiotemporally convergent glutamatergic input to distal dendrites of SPNs are capable of triggering local spikes [11–13,24], as described in pyramidal neurons [41,42]. Importantly, when a ChI-evoked GABAergic PSP was followed a few milliseconds later by dendritic uncaging of glutamate, the SPN response to glutamate was enhanced and often reached the threshold for a local dendritic spike. Thus, from the down-state, GABAergic and glutamatergic synapses worked in concert to drive dendritic depolarization of SPNs.

Simulations of SPN dendritic integration using a biologically accurate NEURON model [13] reproduced this experimental observation, underscoring the importance of dendritic location in determining the interaction of GABAergic and glutamatergic synapses. These studies revealed that the timing of GABAergic inputs became less important to their interaction with glutamatergic synapses as the 2 became more electrotonically remote from one another. Moreover, these simulations suggested that a spatially diffuse, tonic GABA_A_R conductance effectively boosted the response to glutamatergic synaptic signaling over a broad range of values.

### Functional implications for intrastriatal circuitry

The striatum is composed almost entirely of GABAergic neurons, the exception being ChIs. Although there has been a great deal of speculation about the function of the intrastriatal circuitry, its precise role in goal-directed behavior and habit execution remains obscure. In part, this lack of clarity may stem from thinking about GABAergic signaling as being exclusively inhibitory. Our results, in alignment with several previous reports, argue that this narrow view should be broadened. Consider for a moment the role of the intrastriatal GABAergic circuitry controlled by ChIs through fast nAChRs. ChIs have been implicated in several basal ganglia functions, including the response to salient events, set-shifting, and movement sequencing [43]. On the face of it, the robust and diffuse coupling of ChIs to SPNs through “inhibitory” GABAergic interneurons makes no sense in any of these contexts. However, the recognition that ChI-driven GABAergic input to quiescent iSPNs and dSPNs works in concert with glutamatergic signals to promote dendritic depolarization and pull SPNs into an “up-state” creates a much more rational framework. Thus, ChIs serve to rapidly bring SPNs “online” and ready to respond to cortical and thalamic signals directing movement. In this context, it is worth noting that in vivo, SPNs reside well above the K^+^ equilibrium potential [44–46]; this “resting” state appears to be dynamically controlled by synaptic input, which very well could be created by the GABAergic input arising from spontaneously active GABAergic interneurons and those driven by tonically active ChIs [4].

It is also worth considering the potential role of fast-spiking GABAergic interneurons (FSIs). FSIs preferentially target the perisomatic region of SPNs [47] and are widely considered to be part of a fast, feedforward inhibitory circuit linking the striatum with motor cortices [48]. While there is no doubt that FSI input to a spiking SPN is inhibitory, in a quiescent SPN, FSI input should act in precisely the same way as dendritic input and push the membrane potential toward −60 mV. Acting in this way, phasic FSI input to SPNs should facilitate—not inhibit—the response to trailing glutamatergic input from cortical pyramidal neurons. Thus, the timing of signals becomes a critical determinant of whether they should be considered “excitatory” or “inhibitory”; i.e., GABAergic input to SPNs should not be blanketly considered inhibitory.

## Materials and methods

### Animals

All animal experiments were performed according to the NIH Guide for the Care and Use of Laboratory Animals and approved by the Northwestern University Animal Care and Use Committee (approval numbers: IS00019822, IS00016344, IS00010979, and IS00015064 for ASAP, CHDI, JPB, and NIH/NINDS, respectively). Northwestern University has an Animal Welfare Assurance on file with the Office of Laboratory Animal Welfare (A3283-01). The following transgenic male and female mice were used: Adora2a-eGFP (C57BL/6J), RRID:MMRC_010541-UCD; Drd1-tdTomato (FVB), RRID:MMRRC_030512-UNC; Chat-cre (C57BL/6J), RRID:MMRRC_017269-UCD; NPY-cre, (C57BL/6J), RRID:MMRRC_034810-UCD; and Adora2a-cre (C57BL/6J), RRID:MMRRC_034744-UCD. Chat-cre and NPY-cre mice were backcrossed to Adora2a-eGFP and DRD1-tdTomato reporter lines in house. Mice were group-housed with food and water ad libitum on a 12-h light/dark cycle with temperatures of 65° to 75°F and 40% to 60% humidity.

### Stereotaxic surgery

An isoflurane precision vaporizer (Smiths Medical PM) was used to anesthetize mice. Mice were then placed on a stereotaxic frame (David Kopf Instruments), with a Cunningham adaptor (Harvard Apparatus) to maintain anesthesia delivery during surgery. The skull was exposed, and a small hole was drilled at the desired injection site. The following stereotaxic coordinates were used: Striatum, AP = +0.74, ML = −1.85, DV = −3.50. The Allen Mouse Brain Atlas, online version 1, 2008 (RRID:SCR_002978; http://mouse.brain-map.org/static/atlas) was used as a reference for the coordinates and generating diagrams. For each mouse, the distance between bregma and lambda was calculated and used to adjust the coordinates. For AAV injections, approximately 500 nl of viral vector was delivered using a glass micropipette (Drummond Scientific) pulled with a P-97 glass puller (Sutter Instruments). Surgeries for electrophysiology experiments utilizing Chronos were performed unilaterally while surgeries for RiboTag tissue collection were executed bilaterally. Electrophysiology experiments using Chronos were performed after at least 21 postoperative days; tissue collection for RiboTag was performed 10 days after injection (dx.doi.org/10.17504/protocols.io.81wgby191vpk/v1).

### RiboTag profiling

AAVs for expression of RiboTag under a cre-dependent promoter (AAV5-hsyn-DIO-Rpl22l1-3Flag-2A-eGFP-WPRE RRID:Addgene_214265, titers 2.24 × 10^13^ viral genomes/ml) were stereotaxically injected into the striatum in Adora2a-cre mice at P18 or p170, as described above. Ten days after injection, mice were killed and the striatal tissue expressing RiboTag was dissected out using fluorescence microscopy and then frozen at −80°C. RiboTag immunoprecipitation was carried out as previously described [49]. Briefly, tissue was homogenized in cold homogenization buffer [50 mM tris (pH 7.4), 100 mM KCl, 10 mM MgCl_2_, 1 mM dithiothreitol, cycloheximide (100 μg/ml), protease inhibitors, recombinant ribonuclease (RNase) inhibitors, and 1% NP-40]. Homogenates were centrifuged at 10,000*g* for 10 min, and the supernatant was collected and precleared with protein G magnetic beads (Thermo Fisher Scientific) for 1 h at 4°C, under constant rotation. Immunoprecipitations were carried out with anti-Flag magnetic beads (Sigma-Aldrich) at 4°C overnight with constant rotation, followed by 4 washes in high-salt buffer [50 mM tris (pH = 7.4), 350 mM KCl, 10 mM MgCl_2_, 1% NP-40, 1 mM dithiothreitol, and cycloheximide (100 μg/ml)]. RNA was extracted using RNeasy Micro RNA extraction kit (QIAGEN) according to the manufacturer’s instructions (dx.doi.org/10.17504/protocols.io.261gedwyyv47/v1).

### Quantitative real-time PCR

RNA was extracted from the dissected striatal tissue using RNeasy mini kit (QIAGEN). cDNA was synthetized by using the SuperScript IV VILO Master Mix (Applied Biosystems) and preamplified for 10 cycles using TaqMan PreAmp Master Mix and pool of TaqMan Gene Expression Assays (Applied Biosystems). The resulting product was diluted and then used for PCR with the corresponding TaqMan Gene Expression Assay and TaqMan Fast Advanced Master Mix. Data were normalized to *Hprt* by the comparative CT (2-DDCT) method. TaqMan probes were used for PCR amplification of *Hprt*, Mm03024075_m1, Slc12a2 (NKCC1), Mm01265955_m1, Slc12a5 (KCC2), Mm00803929_m1, Slc4a3 (AE3) Mm00436654_g1, and Slc4a10 (NCBE) Mm00473827_m1. Experimental Ct values were normalized to *hprt* values using the following formula: ΔCt = Ct (*gene of interest*) − Ct (*hprt*). The final expression levels were shown as ΔCt values (dx.doi.org/10.17504/protocols.io.e6nvwd4o2lmk/v1).

### Ex vivo slice preparation

Coronal or parasagittal slices (275 μm thickness) were obtained from mice ranging in age from 4 weeks to 9 months. Mice were acutely anesthetized with a mixture of ketamine (50 mg/kg) and xylazine (4.5 mg/kg) and perfused transcardially with oxygenated ice-cold saline (4°C) containing in mM: 125 NaCl, 3 KCl, 2.5 MgCl_2_, 0.5 CaCl_2_, 25 NaHCO_3_, 1.25 NaH_2_PO_4_, and 10 glucose (saturated with 95% O_2_-5% CO_2_; pH 7.4; 300 mOsm/l). After perfusion, mice were decapitated, and the brains were rapidly removed. Slices were obtained in oxygenated ice-cold saline using a vibratome (VT1000S, Leica Microsystems). Slices were transferred to an ACSF-filled holding chamber containing in mM: 125 NaCl, 3 KCl, 1 MgCl_2_, 2 CaCl_2_, 25 NaHCO_3_, 1.25 NaH_2_PO_4_ and 10 glucose (saturated with 95% O_2_-5% CO_2_; pH 7.4; 300 mOsm/l) and held there for approximately 30 min at 34° before being allowed to come to room temperature (21 to 25°C) where they remained until recording (dx.doi.org/10.17504/protocols.io.dm6gp328jvzp/v2).

### Electrophysiological recordings

For electrophysiological recordings slices were transferred to a submersion-style recording chamber mounted on an Olympus BX51 upright microscope (60×/1.0 NA objective) equipped with infrared differential interference contrast. Whole-cell and perforated patch clamp electrophysiological recordings were performed with Multiclamp 700B amplifier. Signals were filtered at 1 KHz. Stimulation and display of electrophysiological recordings were obtained with custom-written freeware *WinFluor* (John Dempster, Strathclyde University, Glasgow, United Kingdom; http://spider.science.strath.ac.uk/sipbs/software_winfluor.htm) that synchronizes two-photon imaging and electrophysiology. Targeted electrophysiological recordings were obtained from visually identified iSPNs or dSPNs. Patch pipettes (3 to 5 MΩ) were prepared with a Sutter Instruments horizontal puller using borosilicate glass with filament and filled with (in mM): 120 potassium-D-gluconate, 13 KCl, 10 HEPES, 0.05 EGTA, 4 ATP-Mg, 0.5 GTP-Na, 10 phosphocreatine-di (tris); pH was adjusted to 7.25 with KOH and osmolarity to 275 to 280 mOsm (dx.doi.org/10.17504/protocols.io.rm7vzx1w2gx1/v2). In perforated-patch experiments, 10 μM gramicidin was added to the internal recording solution to induce chloride-impermeable pore formation along with 25 μM Alexa Fluor 568 hydrazide Na^+^ salt (Invitrogen) to visualize any potential pore rupture. All perforated-patch recordings were corrected for liquid junction potential. Electrophysiological characterization of neurons was made in current clamp configuration. The amplifier bridge circuit was adjusted to compensate for electrode resistance. Access resistances were continuously monitored, and experiments were discarded if changes >20% were observed (dx.doi.org/10.17504/protocols.io.36wgq3y9olk5/v1). Digitized data were imported for analysis with commercial software (IGOR Pro 6.0, WaveMetrics, Oregon RRID:SCR_000325).

### Optogenetic stimulation

Simultaneous electrophysiological and Chronos optogenetic photo-stimulation or RuBi-GABA uncaging were performed with a targeted focal spot blue laser (473 nm Aurora laser launch, Prairie Technologies) system using the *Photostimulus Editor* in *WinFluor*. The Point Photo-activation module (Prairie Technologies) allows 2 different stimulation areas, and intensities, with sub-μm (small spot) critical illumination or an additional lens to stimulate approximately 8 μm (large spot) diameter photo-stimulation in the sample focal plane with the 60×/1.0 objective. To control the release of synaptic GABA, Chat-cre and NPY-cre mice were injected with Chronos (AAV-/hsyn-flex-chronos-GFP or AAV-/hsyn-flex-chronos-tdTomato; UNC GTC Vector Core) as described in the Stereotaxic Surgery section of these methods. To activate NPY or Chat Chronos containing axons in the striatum, the targeted 473 nm spots were positioned adjacent to individual dendritic spines to photo-stimulate presynaptic terminals impinging on iSPNs or dSPNs. The laser power was calibrated to evoke a somatic postsynaptic potential of 2 to 5 mV. Although the laser was aimed peri-dendritically, the blue excitation laser light will travel in a focusing hourglass (small spot) or column (larger spot) through the slice with likely activation of the large dendritic fields of the striatal interneurons, above and possibly below the sample focal plane, resulting in diffuse synaptic GABA_A_R activation of postsynaptic receptors. For simultaneous stimulation of 5 to 10 spines and the reversal potential experiments, the larger blue laser spot was used. Additionally, whole-field photo-stimulation through the 60× objective (26.5FN with approximately 440 μm diameter exposure) was coordinated with an epi-fluorescence-based LED (475/30 nm, pE-100, CoolLED) reflected through an eGFP filter cube and controlled with the *Stimulus Editor* in *WinFluor*. The time synchronized results were displayed in the *WinFluor* main *Record Images and Signals* window (dx.doi.org/10.17504/protocols.io.rm7vzx2nrgx1/v2).

### Two-photon excitation uncaging of DNI-Glutamate

Simultaneous two-photon laser uncaging (720 nm) and optogenetic stimulation of synaptic GABA (473 nm) were performed using a laser scanning microscope system (Ultima, Bruker Technologies; formerly Prairie Technologies) with a tunable imaging laser (Chameleon-Ultra1, Coherent Laser Group, Santa Clara, California, United States of America) and Olympus BX-51WI upright microscope with 60×/1.0NA water-dipping objective lens was used to locate and acquire a whole-cell patch clamp; 810 nm from the imaging 2P laser excited Alexa Fluor 568 (580 to 630 nm; R3896 PMT, Hamamatsu) to visualize dendrites of the patched soma and distal (>100 μm) dendritic spines from planar sections (approximately 20 μm) of the same dendrite with image zoom 4 and 50 μm FOV. Custom written software (*WinFluor*, John Dempster and its *PhotoStimulusEditor* module, Nicholas Schwarz; features now available in *PrairieView* 5.x) was used to direct, control, test, synchronize, and display electrophysiological recordings combined with laser imaging and photo-stimulation. Simultaneous 2PLU (720 nm, Coherent Chameleon) and single-photon optogenetic stimulation of synaptic GABA (473 nm, Prairie Aurora Laser Launch) were provided by a second, separate, independently controlled galvanometer mirror pair in the Ultima system. The 3 laser beams were optically combined (760DCLPXR, Chroma Technologies) in the scan head and aligned to the microscope optical path. DNI-glutamate (5 mM, Femtonics, Budapest, Hungary) was perfused in the recorded area and then excited by the 720 nm 2P laser. Pulses of 1 ms duration (approximately 10 mW sample power) were delivered to single spines located in the same focal plane where the laser average power or spot location was calibrated to evoke a somatic excitatory PSP of 1 to 2 mV for each spine. During synchronized acquisitions, the blue laser GABA photo-stimulation (1 pulse, 3-ms duration) preceded the glutamate uncaging of approximately 15 spines with 1-ms duration and 1-ms inter-stimulation interval. These experiments were all conducted in the appropriate cocktail of synaptic blockers: CGP-55845 (1 μM), MPEP (1 μM), and CPCCOEt (50 μM) (dx.doi.org/10.17504/protocols.io.rm7vzx1w2gx1/v2).

### Pharmacological reagents

Stock solutions were prepared before experiments and added to the perfusion solution or focally applied with pressure ejection in the final concentration indicated. Two-photon laser uncaging and optogenetic experiments were performed in the presence of AP5 (50 μM), NBQX (5 μM), CGP 55845 (1 μM), MPEP (1 μM), and CPCCOEt (50 μM). Bumetanide (10 μM) and acetazolamide (10 μM) were used to probe for roles of NKCC1 and carbonic anhydrase, respectively. All drugs were obtained from Hello Bio, Sigma-Aldrich or Tocris.

### Confocal imaging

Fixed tissue was prepared by transcardially perfusing terminally anesthetized mice with phosphate-buffered saline (PBS; Sigma-Aldrich) immediately followed by 4% paraformaldehyde (PFA; diluted in PBS from a 16% stock solution; Electron Microscopy Sciences). The brain was then removed and transferred into PFA solution overnight before being thoroughly rinsed and stored in PBS at 4°C. Fixed brains were then sectioned into 50-μm thick coronal slices on a Leica VT1200S vibratome and collected in PBS. The sections were positioned on microscopy slides (VWR), allowed to dry and mounted with ProLong Diamond (Thermo Fisher Scientific) and #1.5 glass coverslips (VWR). Mounted sections were stored at 4°C until imaged with an Olympus FV10i-DUC confocal laser scanning microscope, using 10×/0.4 (air) or 60×/1.35 (oil) objective. FIJI (NIH, RRID:SCR_002285) was used to adjust images for brightness, contrast, and pseudo-coloring (dx.doi.org/10.17504/protocols.io.kxygx3nrkg8j/v1).

### Modeling

The NEURON (Neuron 8.2; RRID:SCR_005393) [31] + Python (Python Programming Language RRID:SCR_008394) model of a morphologically reconstructed SPN was integrated into a previously established model [13,29,30]. Cytoplasmic resistivity (Ra) was set to 200 Ω cm and specific capacitance was 1 μF cm^-2^. The original compartmentalized model (https://senselab.med.yale.edu/ModelDB/ShowModel?model=266775&file=/lib/params_dMSN.json#tabs-2) is biophysically detailed and comprised a total of 700 segments with the following active and passive conductances: transient fast inactivating Na^+^ (Naf), persistent Na^+^ (Nap), fast A-type K^+^ (Kaf), slowly inactivating K^+^ (Kas), inwardly rectifying K^+^ (Kir), delayed rectifier K^+^ (Kdr), small conductance Ca^2+^-activated K^+^ (SK), large conductance Ca^2+^-activated K^+^ (BK), L-type Ca^2+^ (Ca_v_ 1.2 and 1.3), N-type Ca^2+^ (Ca_v_ 2.2), R-type Ca^2+^ (Ca_v_ 2.3) T-type Ca^2+^ (Ca_v_ 3.2 and 3.3). Channel distributions over cellular compartments were as previously described and are presented in Table A and Table B in S1 Text (based on Table 2 from Lindroos and colleagues). Synaptic spines were added to all dendritic locations further than 30 μm from the cell soma. Spines were added at a density of 1.711 per μm to give a total of circa 5,500 spines for the reconstructed dSPN. The spines comprised a cylindrical head with a diameter of 0.5 μm connected to dendrites via a neck 1-μm long with diameter of 0.1 μm. The morphologically reconstructed model dSPN had a resting membrane potential of −84 mV; a modest hyperpolarizing current step of 200 pA gave a “rectified range” input resistance of approximately 85 MΩ and membrane time constant of 10.5 ms. The dSPN had an estimated whole-cell capacitance of 180 pF. Candidate spines were selected as separate nearest neighbors along a dendrite at a start point of approximately two-thirds the length. NMDA and AMPA conductances were inserted into spines to be activated. Synaptic currents were modeled using a two-state kinetic model where the normalized peak conductance is determined by rise and decay time constants T_1_ and T_2_ (T_2_ > T_1_) (as per Du and colleagues, Lindroos and colleagues, Lindroos and Kotaleski). The maximal conductances of AMPA and NMDA responses were 350 and 752.5 pS, respectively. The reversal potentials for AMPA and NMDA was 0 mV. Spines were activated at 1 ms intervals in succession with stimulation moving away from the soma as per electrophysiological activation. The threshold for generating an up-state in the absence of any GABAergic activation was 15 glutamatergic inputs. GABA synapses were inserted directly onto the same dendritic location (the midpoint of the chosen dendrite). The maximal conductance was 1,000 pS and reversal potential set to −60 mV. GABAergic responses were generated by activating these synapses simultaneously in up to groups of 3 at an interval of 1 ms. For most simulations, onsite activation comprised a total of 12 activated GABA synapses. For offsite activation, 4 distal locations were selected and activated simultaneously multiple times at an interval of 1 ms. Again, for most simulations each dendrite received a burst of 3 activated GABA synapses at an interval of 1 ms making a total of 12 activated GABA synapses across 4 dendritic locations. All code for the simulations is publicly available (https://github.com/vernonclarke/SPNfinal/tree/v1.0; dx.doi.org/10.5281/zenodo.10162265).

### Statistical analysis

Data was graphically presented using nonparametric box and whisker plots. In these plots, the center line is the median, the edges of the box mark the interquartiles of the distribution and the lines extend to the limiting values of the sample distribution; outliers (defined as values further away from the median than 1.5 × interquartile range) are marked as asterisks. Statistical significance was determined using nonparametric tests (Wilcoxon signed rank test using either the exact method or with continuity correction and Mann–Whitney U test, as appropriate) using R Statistical Software (v4.2.3; R Core Team 2023, RRID:SCR_001905) [50]. R was used to perform linear regression analysis of data used to estimate reversal potentials. The regression analysis was performed with mean values for data at each voltage. Means were rounded to the nearest whole number for the current traces (Fig 2B and 2C) and to the nearest 0.5 mV for the ΔV/V traces (Fig 2E and 2F) prior to running the regression.

## Supporting information

S1 Fig The summation of GABAergic and glutamatergic PSPs was typically linear or supralinear. **(A)** Diagram showing how the amplitudes of the individual ChI-evoked GABAergic and 2P uncaging-evoked glutamatergic PSPs were measured, along with the amplitude of the response to combined stimulation (see Fig 4). **(B)** Scatter plot of response amplitude to the combined stimulation against the arithmetic sum of the individual GABAergic and glutamatergic PSPs. Most of the data points derived from individual SPNs fell on the diagonal or above, demonstrating linearity or supralinear behavior. The data underlying the graphs shown in the figure can be found in dx.doi.org/10.5281/zenodo.10387118.
(EPS)

S2 Fig Reducing cytoplasmic resistivity (R_a_) has no effect on the fundamental outcomes of computational modeling of dSPN dendritic activity. The simulations in this figure are identical to those of Fig 5, except cytoplasmic resistivity (R_a_) is reduced from 200 to 100 Ω cm and the underlying conductances of synaptically activated glutamatergic AMPA and NMDARs are increased by 60% (from 350 and 752.5 to 560 and 1,204 pS, respectively). The latter ensures that a glutamatergic synaptic event at a given spine produces a similar depolarization at its equivalent dendritic location (i.e., as measured in the dendritic tree at the location of its spine neck). As a result, the number of clustered inputs that was just subthreshold for upstate generation (15) was preserved. Any qualitative differences (small increases in “quarterdrop duration” (B and C) and somatic amplitude (B, C, F, and I) can be attributed to the effect of reduced dendritic filtering resulting from reduced R_a_. The data underlying the graphs shown in the figure can be found in dx.doi.org/10.5281/zenodo.10387118.
(EPS)

S3 Fig Temporal profile of interaction between glutamatergic and GABAergic synaptic activity. **(A)** On-path phasic GABAergic activity is delivered to a dendrite positioned more proximally on the same path to the soma as the dendrite receiving glutamatergic synaptic input. As before, synaptic potentials are illustrated at **(B)** dendritic site of glutamatergic activity and **(C)** cell soma. L-glutamate mediated excitation alone (15 neighboring spines activated at 1 ms intervals along the chosen dendrite; black trace), phasic GABAergic stimulation alone (3 simultaneous synaptic stimulations delivered 4 times at an interval of 1 ms to the proximal on-path dendritic site giving a total of 12 GABA synapses activated; light gray) and the effect of varying glutamatergic stimulation at temporal intervals relative to this fixed GABAergic input described (blue to orange illustrate 5 traces with Δ**t** = t_GLUT_−t_GABA_ in the range of −10 to 30 ms, respectively, at 10 ms intervals) are illustrated. The absolute amplitude of the synaptic potential in the dendrite was measured in the absence of GABAergic activity (P_1_) or either relative to the amplitude of the underlying depolarizing phasic GABAergic potential response (P_2_) or relative to the underlying baseline (P_3_). **(D)** and **(E)** show the supralinear effect of varying glutamatergic spine activation relative to a fixed distal off-path GABA synaptic input on P_2_ and P_3_ normalized to P_1_, respectively. For comparison, data from the off-path distally located phasic GABAergic activity is overlayed (D and E). The widened temporal window for supralinear summation in this simulation arises from the reduced path length from the site of GABA activation to the site of glutamatergic clustered activity which manifests as an increase in magnitude of GABAergic PSP at this location (e.g., compare GABAergic PSPs; light gray trace in panel B to its equivalent in Fig 6G). The data underlying the graphs shown in the figure can be found in dx.doi.org/10.5281/zenodo.10387118.
(EPS)

S4 Fig NMDAR dependence and GABAergic threshold for dendritic spike generation. **(A)** Offsite phasic GABAergic activation delivered to 4 distal dendritic locations with the same clustered glutamatergic input (15 spines; 1 ms interval) delivered to the dendrite as in Fig 5G but with NMDAR conductance set to zero. This resulted in a synaptic potential (red) at the dendritic site of delivery **(B)** and soma **(C)**. Similarly, the effect of only activating GABAergic synapses simultaneously at each of the 4 offsite dendritic locations (3 per dendrite at 1 ms intervals; 12 in total) resulted in a moderate postsynaptic potential (blue trace). When delivered sequentially, with GABAergic activation preceding glutamatergic by 10 ms, the previously subthreshold glutamatergic input did not result in the generation of an up-state (black trace). Thus, supralinear summation to phasic GABAergic input is dependent on the synaptic activation of NMDARs. **(D)** Offsite phasic GABAergic activation delivered to the midpoint of 12 most distal dendritic locations. As before, synaptic potentials are illustrated at **(E)** dendritic site of glutamatergic activity and **(F)** cell soma. As before, L-glutamate mediated excitation alone (15 neighboring spines activated at 1 ms intervals along the chosen dendrite; black trace), phasic GABAergic stimulation alone (single synaptic stimulations delivered simultaneously to the 12 most distal dendrites giving a total of 12 GABA synapses activated; light gray) and the effect of varying glutamatergic stimulation at temporal intervals relative to this fixed GABAergic input described (blue to orange illustrate 5 traces with Δ**t** = t_GLUT_−t_GABA_ in the range of −10 to 30 ms, respectively, at 10 ms intervals) are illustrated. **(G)** and **(H)** show the supralinear effect of varying glutamatergic spine activation relative to a fixed off-path distal GABA synaptic inputs on P_2_ and P_3_ normalized to P_1_, respectively. For comparison, data from the off-path distally located phasic GABAergic activity with 4 sites each receiving 3 inputs at 1 ms intervals delivered simultaneous across the 4 dendritic locations is overlayed (taken from Fig 6I and 6J). In this example, the widened temporal window for supralinear summation in this simulation most likely arises from the temporal differences in activation (12 delivered simultaneously at 12 sites vs. 12 delivered as a burst of 3 at 1 ms intervals at 4 sites simultaneously; compare GABAergic PSPs: light gray trace in panel E to its equivalent in Fig 6G). This manifests as an increased GABAergic potential at the glutamatergic site of clustered excitation. This simulation confirms that the observed effect of phasic offsite GABAergic activity can be extended to simultaneous activation at many dendritic locations and provides a simplified model to examine the threshold activity required (as it allows synapse number to be varied in a unitary fashion). Synaptic potentials are illustrated at **(I)** dendritic site of glutamatergic activity and **(J)** cell soma for GABAergic activity delivered simultaneously to the midpoint of either 8 and 9 (offset for clarity) most distal dendritic locations. The threshold number of GABAergic synapses necessary for dendritic up-state generation in this model was 9. The data underlying the graphs shown in the figure can be found in dx.doi.org/10.5281/zenodo.10387118.
(EPS)

S1 Text Model parameters and ion channel distributions. **Table A** provides the maximum conductance for all cation channels in the model (S/cm^2^). **Table B** gives the maximum permeability of Ca^2+^ channels in the model (cm/s). The equations are identical to that provided in the original modeling code (Du and colleagues, Lindroos and colleagues, Lindroos and Kotalseki) and retain the original variable names for generating the following distributions: sigmoidal: (a_4_+a_5_/(1+exp((x-a_6_)/a_7_))) *g_max_; exponential: (a_4_+a_5_*exp((x-a_6_)/a_7_)) *g_max_; uniform: g_max_. The initial dendrite measurement is taken at x = 6.010 μm (i.e., the radius of the soma; x is measured from center of cell soma); final dendrite values is taken at x = 265.268 μm which is the path length of the furthest dendritic point from the mid-point of the soma.
(DOCX)

## Abbreviations

2PLSM: two-photon laser scanning microscopy
AAV: adeno-associated virus
CA: carbonic anhydrase
ChAT: choline acetyltransferase
ChI: cholinergic interneuron
FSI: fast-spiking GABAergic interneuron
iGluR: ionotropic glutamate receptor
LTSI: low-threshold spike GABAergic interneuron
nAChR: nicotinic acetylcholine receptor
NGFI: neurogliaform interneuron
NMDAR: N-methyl-D-aspartate receptor
PBS: phosphate-buffered saline
PFA: paraformaldehyde
PSP: postsynaptic potential
qPCR: quantitative polymerase chain reaction
SPN: spiny projection neuron
THI: tyrosine hydroxylase interneuron
