# Correction: GABAergic regulation of striatal spiny projection neurons depends upon their activity state

**DOI:** 10.1371/journal.pbio.3002752

**Published:** 2024-07-26

**Authors:** Michelle Day, Marziyeh Belal, William C. Surmeier, Alexandria Melendez, David Wokosin, Tatiana Tkatch, Vernon R. J. Clarke, D. James Surmeier

In Figs 3 and 4, there is an error in panel E. The inset cartoon in Fig 3E is incorrectly labelled as NGFi/THi. It should show optogenetic activation of NGFIs and LTSIs and be labelled as NGFi/THi and the cholinergic interneuron (ChI) part should have been removed.

**Fig 3:**
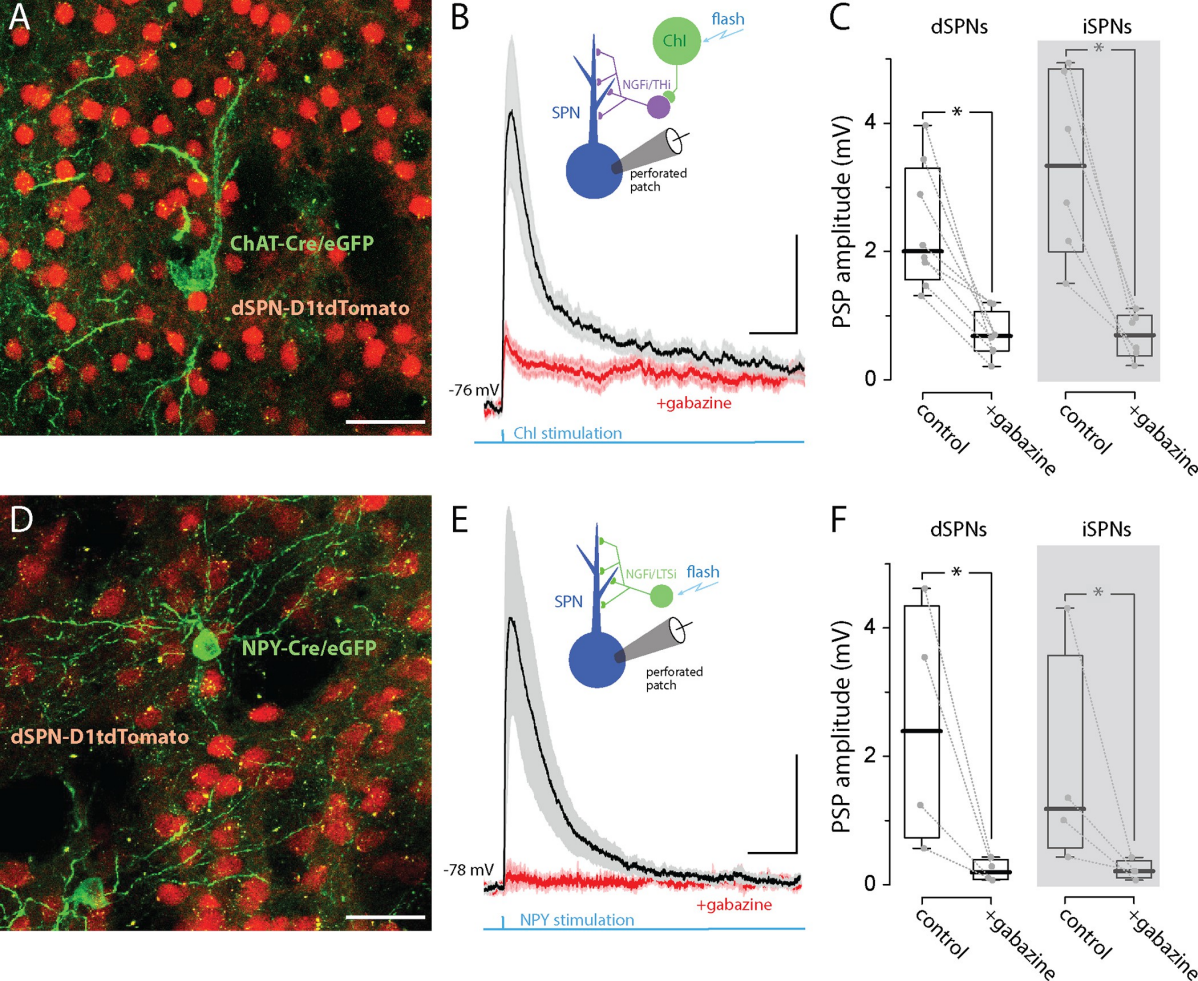
Optogenetic stimulation of ChIs or NPY-expressing interneurons evokes robust GABA_A_R-mediated PSPs in both iSPNs and dSPNs. **(A)** AAV9-hSyn-chronos-flex-eGFP was stereotaxically injected into the striatum of two-month-old ChAT-Cre X D1tdTomato mice (Stereotaxic coordinate injection: ML = −1.2, AP = −0.7, DV = −3.4). The coronal confocal slice image shows the expression of Chronos (green cells) in a ChAT-cre neuron (cholinergic interneurons) along with dSPNs expressing tdTomato (red cells, scale bar = 40 μm). The tissue was dissected and recorded from 21 days postinjection. **(B)** The mean (± SEM) of ChI-evoked EPSP responses recorded from visually identified SPNs in gramicidin perforated patch in current-clamp mode in the presence of synaptic blockers: NBQX (5 μM), AP5 (50 μM), CGP-55845 (1 μM), MPEP (1 μM), and CPCCOEt (50 μM). The LED pulse (470 nm, 5 ms) was applied at an interval of 60 s. The traces recorded before and after the addition of gabazine (10 μM). Scale bars = 1 mV/100 ms. **(C)** Box plots of data from dSPNs (*n* = 8) and iSPNs (*n* = 6). **(D)** NPY-Cre X D1tdTomato mice were injected as described in (A). Confocal image showing NPY-Cre neurons expressing Chronos (green) and dSPNs expressing tdTomato (red, scale bar = 40 μm). **(E)** Mean (+ SEM) of NPY-Cre-evoked EPSP responses recorded from visually identified dSPNs in gramicidin perforated patch in current-clamp mode in the presence of blockers as described in (B) before and after the addition of Gabazine (10 μM). Traces from dSPN recorded in NPY (*n* = 4). Scale bars = 1 mV/100 ms. **(F)** Summary data for dSPNs (*n* = 4) and for iSPNs (*n* = 4). The data underlying the graphs shown in the figure can be found in dx.doi.org/10.5281/zenodo.10386854. ChAT, choline acetyltransferase; ChI, cholinergic interneuron; PSP, postsynaptic potential; SPN, spiny projection neuron.

**Fig 4:**
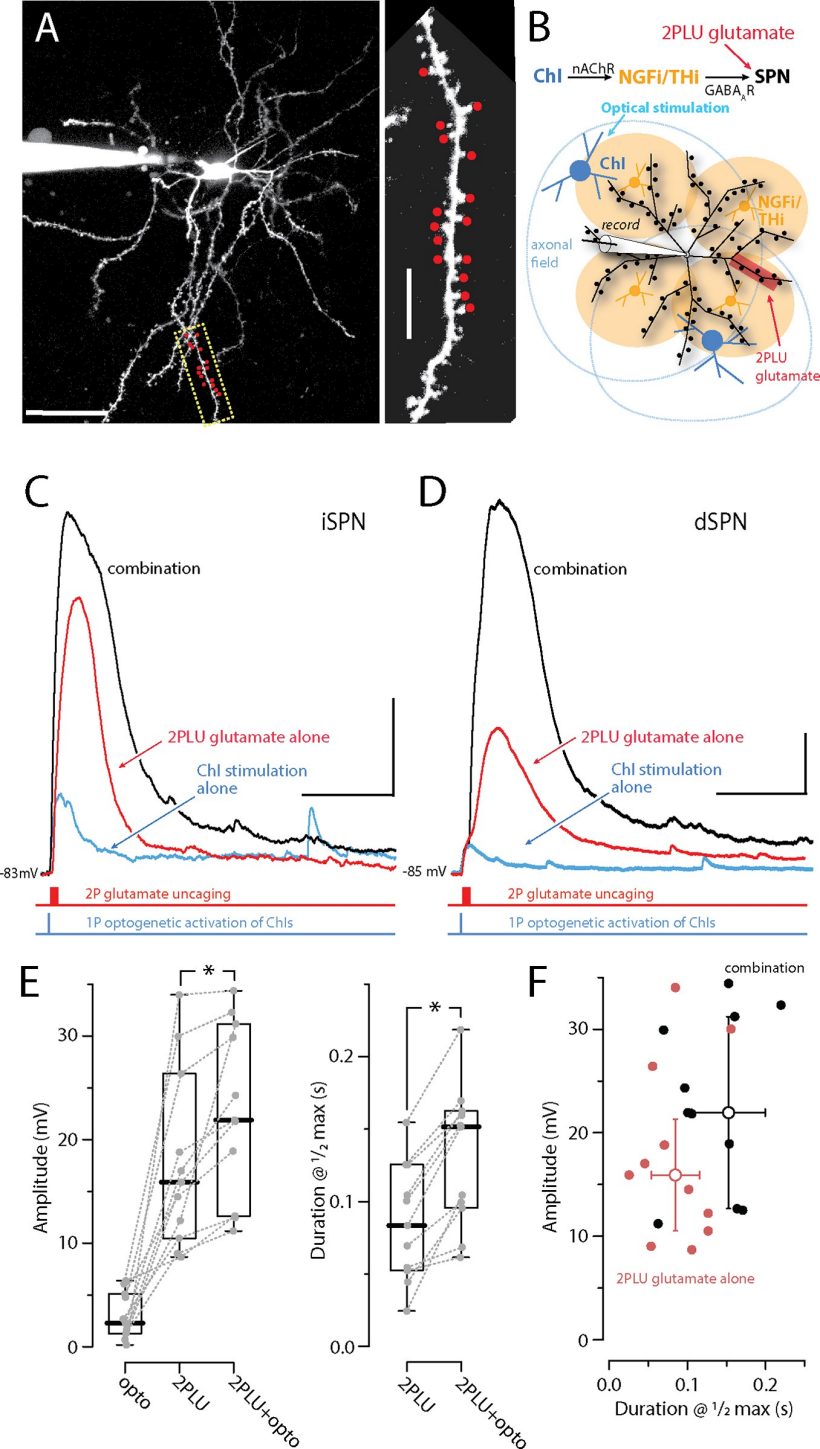
ChI-evoked stimulation of NGF interneurons and synaptic GABA release enhances glutamate-evoked state transitions. **(A)** Maximum projection image of a visually identified dSPN from a D1R-tdTomato x ChAT-cre mouse with a high magnification image of a distal dendrite where 720 nm 2PLSM spot uncaging of DNI-Glu (2PLU, 5 mM) was conducted (red dots). Tomato+ dSPNs were patched in whole-cell mode and the cells were loaded with Alexa 568 for clear identification of dendrites and spines. Scale bars = 40 μm cell, 5 μm dendrite. **(B)** Scheme for interrogating endogenous GABA release from NGFIs onto SPNs via optogenetic stimulation of ChAT-cre mice expressing Chronos. **(C, D)** Throughout the dendrites, glutamate uPSPs in dSPNs and iSPNs can be evoked by uncaging DNI-Glu (5 mM, 1 × 15 spines, 1 ms pulses at 500 Hz, red traces, 720 nm laser) while stimulating GABA release from NGFIs with the blue laser (1 × 3 ms pulse, blue traces, 473 nm, within approximately 20 μm of the dendrite). From the quiescent down-state, GABA_A_R activation is depolarizing and pushes SPNs toward enhanced dendritic integration in both dSPN and iSPN dendrites (Glu-2PLU + GABA_A_ opto = black trace, scale bars = 5 mV/200 ms). **(E)** Summary data showing the enhancement in amplitude and duration of the plateaus at ½ the maximum amplitude (1/2max) in iSPNs and dSPNs combined (*n* = 11 total: 3 iSPNs + 8 dSPNS; *p* **< 0.001** for both amplitude and 1/2max duration, respectively). **(F)** Scatter plot of duration at ½ maximum amplitude vs. amplitude for clustered glutamate alone (red) and following GABA_A_R activation (black). Median effects (open circles) and the median absolute difference as capped lines are also illustrated. All experiments are conducted in the appropriate cocktail of synaptic blockers: CGP-55845 (1 μM), MPEP (1 μM), and CPCCOEt (50 μM). The data underlying the graphs shown in the figure can be found in dx.doi.org/10.5281/zenodo.10386854. 2PLSM, two-photon laser scanning microscopy; ChAT, choline acetyltransferase; ChI, cholinergic interneuron; SPN, spiny projection neuron.

In Fig 4E, the y-axis of the right panel is mislabeled as ‘0.2 and 0.3’. It should have been ‘0.1 and 0.2’. Please see the correct version of Figs 3 and 4 here.

The Data Availability statement for this paper is incorrect. The correct statement is: All datasets are publicly available: Figs 1–8: dx.doi.org/10.5281/zenodo.10386854. S1–S4 Figs: dx.doi.org/10.5281/zenodo.10387118. R code for graphical outputs and statistical analyses of Figs 1–4: https://dx.doi.org/10.5281/zenodo.10386496. Modelling code and R code to recreate all modelling figures https://dx.doi.org/10.5281/zenodo.10162264.
