# Supplementary material for: GABAergic regulation of striatal spiny projection neurons depends upon their activity state
Source: PLoS Biol. 2024 Jan 31;22(1):e3002483. doi: 10.1371/journal.pbio.3002483 (PMC10830145; doi:10.1371/journal.pbio.3002483)
Supplement: S1 Text — Table A provides the maximum conductance for all cation channels in the model (S/cm2). Table B gives the maximum permeability of Ca2+ channels in the model (cm/s). The equations are identical to that provided in the original modeling code (Du and colleagues, Lindroos and colleagues, Lindroos and Kotalseki) and retain the original variable names for generating the following distributions: sigmoidal: (a4+a5/(1+exp((x-a6)/a7))) *gmax; exponential: (a4+a5*exp((x-a6)/a7)) *gmax; uniform: gmax. The initial dendrite measurement is taken at x = 6.010 μm (i.e., the radius of the soma; x is measured from center of cell soma); final dendrite values is taken at x = 265.268 μm which is the path length of the furthest dendritic point from the mid-point of the soma. (DOCX) [file pbio.3002483.s005.docx]

**S1 Text**

**Table A:** Max conductance of monovalent cation channels in the model (S/cm2)

| Name | axon | soma | initial dendrite | final dendrite | spine neck | spine head | dendritic distribution | dendritic variables |
| --- | --- | --- | --- | --- | --- | --- | --- | --- |
| NaF | 9 | 6 | 6.9150e-1 | 3.1342e-10 | 0 | 0 | sigmoidal | a_4_=0; a_5_=1; a_6_=50; a_7_=10; g_max_=0.7 |
| NaP | 0 | 7e-4 | 0 | 0 | 0 | 0 | NA | NA |
| KaF | 0 | 1.5e-1 | 8.9343e-2 | 6.0235e-2 | 0 | 0 | sigmoidal | a_4_=0.5; a_5_=0.25; a_6_=120; a_7_=30; g_max_=0.12 |
| KaS | 7e-3 | 1.6e-2 | 3.5896e-2 | 3.0e-3 | 0 | 0 | exponential | a_4_=0.25; a_5_=5; a_6_=0; a_7_=-10; g_max_=1.2e-2 |
| Kdr | 0 | 9.4e-4 | 7.4375e-4 | 1.7554e-4 | 0 | 0 | sigmoidal | a_4_=0.25; a_5_=1; a_6_=50; a_7_=30; g_max_=7e-4 |
| Kir | 0 | 1.2e-4 | 2.4e-4 | 2.4e-4 | 0 | 1e-7 | linear | g_max_=2.4e-4 |
| BK | 0 | 1.3e-4 | 1e-4 | 1e-4 | 0 | 0 | linear | g_max_=1e-4 |
| SK | 0 | 2e-5 | 2e-5 | 2e-5 | 0 | 2e-5 | linear | g_max_=2e-5 |
| e_pas_ | 1.25e-5 | 1.25e-5 | 1.25e-5 | 1.25e-5 | 1.25e-5 | 1.25e-5 | linear | g_max_=1.25e-5 |
| I_m_ | 1e-3 | 0 | 0 | 0 | 0 | 0 | NA | NA |

**Table B:** Max permeability of Ca^2+^ channels in model (cm/s)

| Name | axon | soma | initial dendrite | final dendrite | spine neck | spine head | dendritic distribution | dendritic variables |
| --- | --- | --- | --- | --- | --- | --- | --- | --- |
| Ca_v_ 1.2 L-type | 0 | 1.34e-5 | 1e-5 | 1e-5 | 0 | 1e-7 | uniform | p_max_=1e-5 |
| Ca_v_ 1.3 L-type | 0 | 1.34e-6 | 1e-6 | 1e-6 | 0 | 1e-6 | uniform | p_max_=1e-6 |
| Ca_v_ 3.2 T-type | 0 | 0 | 4.1766e-7 | 9.9596e-6 | 1e-7 | 1e-7 | sigmoidal | a_4_=0; a_5_=1; a_6_=100; a_7_=-30; p_max_=1e-5 |
| Ca_v_ 3.3 T-type | 0 | 0 | 1.0442e-7 | 2.4899-6 | 1e-7 | 1e-8 | sigmoidal | a_4_=0; a_5_=1; a_6_=100; a_7_=-30; p_max_=2.5e-6 |
| Ca_v_ 2.2 N-type | 0 | 4e-5 | 1e-7 | 1e-7 | 0 | 0 | linear | p_max_=1e-7 |
| Ca_v_ 2.3 R-type | 0 | 1.34e-4 | 5e-4 | 5e-4 | 0 | 0 | linear | p_max_=5e-4 |
